# Symptom-based early-stage differentiation between SARS-CoV-2 versus other respiratory tract infections—Upper Silesia pilot study

**DOI:** 10.1038/s41598-021-93046-6

**Published:** 2021-06-30

**Authors:** Justyna Mika, Joanna Tobiasz, Joanna Zyla, Anna Papiez, Małgorzata Bach, Aleksandra Werner, Michał Kozielski, Mateusz Kania, Aleksandra Gruca, Damian Piotrowski, Barbara Sobala-Szczygieł, Bożena Włostowska, Paweł Foszner, Marek Sikora, Joanna Polanska, Jerzy Jaroszewicz

**Affiliations:** 1 Department of Data Science and Engineering, Silesian University of Technology, Gliwice, Poland; 2 Department of Applied Informatics, Silesian University of Technology, Gliwice, Poland; 3 Department of Computer Networks and Systems, Silesian University of Technology, Gliwice, Poland; 4 Department of Graphics, Computer Vision and Digital Systems, Silesian University of Technology, Gliwice, Poland; 5 Department of Infectious Diseases and Hepatology, Medical University of Silesia, Katowice, Poland

**Keywords:** Epidemiology, Respiratory signs and symptoms, Risk factors

## Abstract

In the DECODE project, data were collected from 3,114 surveys filled by symptomatic patients RT-qPCR tested for SARS-CoV-2 in a single university centre in March-September 2020. The population demonstrated balanced sex and age with 759 SARS-CoV-2( +) patients. The most discriminative symptoms in SARS-CoV-2( +) patients at early infection stage were loss of taste/smell (OR = 3.33, *p* < 0.0001), body temperature above 38℃ (OR = 1.67, *p* < 0.0001), muscle aches (OR = 1.30, *p* = 0.0242), headache (OR = 1.27, *p* = 0.0405), cough (OR = 1.26, *p* = 0.0477). Dyspnea was more often reported among SARS-CoV-2(-) (OR = 0.55, *p* < 0.0001). Cough and dyspnea were 3.5 times more frequent among SARS-CoV-2(-) (OR = 0.28, *p* < 0.0001). Co-occurrence of cough, muscle aches, headache, loss of taste/smell (OR = 4.72, *p* = 0.0015) appeared significant, although co-occurrence of two symptoms only, cough and loss of smell or taste, means OR = 2.49 (*p* < 0.0001). Temperature > 38℃ with cough was most frequent in men (20%), while loss of taste/smell with cough in women (17%). For younger people, taste/smell impairment is sufficient to characterise infection, whereas in older patients co-occurrence of fever and cough is necessary. The presented study objectifies the single symptoms and interactions significance in COVID-19 diagnoses and demonstrates diverse symptomatology in patient groups.

## Introduction

One of the biggest challenges connected with COVID-19 pandemic is the ability of healthcare service to provide every patient in need with required health care. Limited testing capacity, lack of highly effective treatment and maximum hospital load are a bottleneck of effective pandemic overcoming. Due to the above, the ability to distinguish patients with and without COVID-19 among symptomatic patients at the early stage of disease appears to play a crucial role.

The typical manifestation of SARS-CoV-2 infection is related to its pathophysiology. The virus strikes mostly by aerosol transmission, is inhaled into the respiratory tract, and therefore the disease primarily affects the lungs^1^. The course of the infection vary from asymptomatic (no clinical manifestations, no changes in CT or X-ray scans, positive RT-qPCR for SARS- CoV-2), mild (typical and atypical clinical symptoms, lack of manifestations of pneumonia), moderate (manifested as a pneumonia both clinically and radiologically), severe (dyspnea, hypoxia, pneumonia involving > 50% of lungs), and critical (with ARDS (acute respiratory distress syndrome) or respiratory failure, myocardial injury, arrhythmia, or heart failure, AKI (acute kidney injury), acute liver injury, encephalopathy, DIC (disseminated intravascular coagulation), rhabdomyolysis, septic shock, or multiple organ dysfunction)^2^. The spectrum of possibilities of how a certain patient would undergo the disease and similarities to the course of other breathable pathogens makes the quick diagnosis of SARS-CoV-2 infection crucial. After almost a year since the disease has spread from China, the typical spectrum of symptoms of SARS-CoV-2 infection has been observed. In a typical case, a patient will suffer from dry cough and high fever, when viral pneumonia develops, it will result in shortness of breath^3–5^. Additionally, muscle ache, confusion, headache, sore throat, chest pain, diarrhoea, nausea/vomiting, conjunctival congestion, fatigue, smell or taste disorders need also to be pointed out as symptoms related to SARS-CoV-2 infection^3,4,6–9^.

To confirm SARS-CoV-2 infection, viral nucleic acid or antigen detection is required. Since the result of PCR testing for SARS-CoV-2 usually is not available within minutes, the decision whether a patient should be isolated or requiring quarantine needs to be made before, often soon after specimen collection. Admittedly, novel generation SARS-CoV-2 antigen tests provide a rapid diagnosis in highly viraemic, symptomatic patients with high sensitivity ≥ 90% and specificity ≥ 97%^10^. On the other hand, it is crucial to remember that sensitivity of these tests could be substantially lower in asymptomatic individuals, and interpretation of results should take into consideration not only analysis of symptoms but also local SARS-CoV-2 prevalence^11^.

The similarities of symptoms that can be found in SARS-CoV-2 infected patients and symptoms in patients with respiratory tract infection caused by other agent was also noticed by Alpaydin et al.^12^ in their study of 112 patients with respiratory tract infection hospitalised in March 2020, where SARS-CoV-2 infection was confirmed in 30% of cases, remaining cases were caused by metapneumovirus, rhinovirus, adenovirus, respiratory syncytial virus, influenza virus and *Mycoplasma pneumoniae*. Recent studies in Brazil performed on over 6000 patients show various single symptoms characteristic for SARS-CoV-2, e.g. fever, cough, breathing difficulty or diarrhoea, just to mention a few^13^. This work concentrates mainly on building classification system and does not include co-occurrence analysis and differences within age and sex. Another research^14^ focused primarily on understanding the loss of taste and smell during SARS-CoV-2 infection. Makaronidis et al. conducted a study on 590 patients, which shows that loss of smell and/or taste might occur without typical symptoms like cough or fever. Moreover, other studies characterise patients' prognosis regarding the comorbidities occurrence^15^. Kolhe et al.^16^ report a high incidence of AKI in patients with COVID-19 that was associated with a threefold higher odds of death than COVID-19 without AKI and a fourfold higher odds of death than AKI due to other causes. Rentsch et al.^17^ and Harrison et al.^18^ show that mortality due to SARS-CoV-2 is related to age, sex, ethnicity, comorbidities. Finally, many studies focus on analysing COVID-19 symptoms based on population in certain regions only^13,19–23^. As the research results show^24^, the course of the disease for various reasons may be different for different populations, and therefore, the analysis of data from Poland may broaden knowledge about the characteristics of the COVID-19 pandemic.

The recently published results of meta-analysis^25^ demonstrated that based on currently available data, neither absence nor presence of signs or symptoms are accurate enough to rule in or rule out COVID-19. The presence of anosmia or ageusia may be useful as a red flag for COVID-19. The presence of fever or cough, given their high sensitivities, may also be useful to identify people for further testing. The paper also agreed that further studies in an unselected population presenting to primary care or hospital outpatient settings, examining combinations of signs and symptoms to evaluate the syndromic presentation of COVID-19, are still urgently needed.

As stated in^25^, only two studies (Gilbert et al.^26^; Yombi et al.^27^) assessed combinations of different signs and symptoms. Gilbert et al. investigated six combinations of two to four symptoms each, while Yombi et al. considered three combinations of two to three symptoms each. Most of the combinations included fever and cough, on which both studies had preselected their participants. These combinations led to specificities above 80%, but at the cost of low sensitivities (< 30%). The aim of this research was to analyse the occurrence and co-occurrence of SARS-CoV-2 infection early symptoms against a control group comprising individuals who report to a medical facility to be tested, but show to be SARS-CoV-2 negative. The identification of a set of symptoms which co-occurrence yields the strongest discrimination between SARS-CoV-2 positive and negative individuals (e.g., among younger and older, men and women) was a particularly relevant finding.

## Results

### Demographic and epidemiological profile

3,114 patients filled the surveys (Table 1). Among them, about 25% were tested positive for SARS-CoV-2 infection, whereas the remaining 75% got negative test results. The population was balanced regarding sex. Among all patients, 62% reported symptoms of infection and the remaining 38% were categorised as healthy. The age of patients ranged from 1 to 98 years, with a median of 42. 1,201 people were aware of their blood type, among which the most common were type 0 (39%) and type A (34%), and the least common was type AB (almost 10%). Detailed characteristics of the sample are provided in Supplementary Table S1.

**Table 1:** Summary of data based on demographic profile. One patient did not provide information regarding sex.

	**SARS-CoV-2 ( +)**	**SARS-CoV-2 (-)**	**ALL**
	(**n**/% of N)	(**n**/% of N)	(**N**/% of *N.ALL*)
All	**759**/24.37%	**2355**/75.63%	*N.ALL* = **3114**/100%
**Sex**			
Men	**408**/26.17%	**1151**/73.83%	**1559**/50.08%
Women	**351**/22.59	**1203**/77.41%	**1554**/49.92%
**Symptoms occurrence**			
Symptomatic	**586**/30.19%	**1355**/69.81%	**1941**/62.33%
Asymptomatic	**173**/14.75%	**1000**/85.25%	**1173**/ 37.67%

### Demographic profile of symptomatic patients

First, we investigated the completeness of the surveys in the subset of 1,941 symptomatic patients. About half of the queries had more than 30% missing information, including five characteristics that did not have answers provided by more than 60% of patients. The other half of the queries had information provided by all patients (10 characteristics) or by most of the patients (4 queries). Supplementary Figure S1 shows the distribution of missing values. Next, we examined the demographic and epidemiological profile of symptomatic patients regarding the COVID-19 diagnosis versus other respiratory tract infections. Among 1,941 patients presenting any symptoms, 30% (n = 586) were infected with SARS-CoV-2, and the remaining 70% (n = 1355) got negative test results (Table 1). There were no statistically significant differences, neither in the number of men/women with and without COVID-19 nor the relation between the age of patients and their diagnosis (Table 2). Noteworthy, pairwise analysis of age and sex regarding COVID-19 diagnosis showed significant differences in median age between men and women in the group of people infected with SARS-CoV-2 (*p* = 0.0140). Women get a positive diagnosis of COVID-19 when being older compared to men, however, with negligible effect (OR = 1.01). Blood type was evenly distributed in both groups of patients. As expected, having contact with an infected person significantly twofold increased the odds of being SARS-CoV-2 infected (Table 3). The duration of symptoms is slightly longer for people not infected with SARS-CoV-2, however careful examination of results showed that the effect is driven by people reporting many days of observing symptoms (> 10) in the group of COVID-19 negative diagnosis. The median duration of symptoms in both groups equals four days.

**Table 2:** The differences in prevalence of studied clinical characteristics between patients positive and negative for SARS-CoV-2.

Clinical characteristic	N (SARS-CoV-2 (−) / SARS-CoV-2 ( +))*	SARS-CoV-2 (-) Mean value ± SD	SARS-CoV-2 ( +) Mean value ± SD	OR (95% CI)	OR effect interpretation	OR *p*-value ***
Age (years)	1938 (1354/584)	42.58 ± 14.68	43.34 ± 13.30	1.00 (1.00–1.01)	Negligible	0.2826
Baseline Sp02 w/o oxygen supplementation (%)	1887 (1312/575)	97.19 ± 1.27	97.05 ± 1.38	0.93 (0.86–1.00)	Negligible	0.0400
Temp. in hospital [^o^C]	1849 (1282/567)	36.51 ± 0.46	36.50 ± 0.51	0.98 (0.79–1.20)	Negligible	0.8094

Clinical characteristic (Yes)	N (SARS-CoV-2 (-) / SARS-CoV-2 ( +))	SARS-CoV-2 (-) N (%)*	SARS-CoV-2 ( +) N (%)*	OR (95% CI)	OR effect interpretation	OR* p*-value***
Sex **	1940 ι(1354/586)	F: 680 (50.22%)	F: 273 (46.59%)	0.87 (0.71–1.05)	Negligible	0.1416
Allergies	1941 (1355/586)	47 (3.47%)	8 (1.37%)	0.46 (0.23–0.91)	Small	0.0264
Heart diseases	1941 (1355/586)	89 (6.57%)	24 (4.10%)	0.64 (0.41–1.00)	Small	0.0515
Lung disease	1941 (1355/586)	29 (2.14%)	4 (0.68%)	0.44 (0.18–1.05)	Small	0.0655
Asthma	1941 (1355/586)	71 (5.24%)	20 (3.41%)	0.68 (0.42–1.11)	Small	0.1219
Diabetes	1941 (1355/586)	71 (5.24%)	22 (3.75%)	0.75 (0.46–1.20)	Negligible	0.2227
Hypertension	1941 (1355/586)	250 (18.45%)	100 (17.06%)	0.91 (0.70–1.17)	Negligible	0.4662
Kidney diseases	1941 (1355/586)	17 (1.25%)	6 (1.02%)	0.96 (0.42–2.22)	Negligible	0.9313

Blood type	N(SARS-CoV-2 (-) / SARS-CoV-2 ( +))	SARS-CoV-2 (-) N (% of 477)	SARS-CoV-2 ( +) N (% of 256)	OR (95% CI)	OR effect interpretation	OR* p*-value***
0	292 (196/96)	196 (41.09%)	96 (37.50%)	Serving as reference		
A	260 (162/98)	162 (33.96%)	98 (38.28%)	1.24 (0.87–1.75)	Negligible	0.2371
B	112 (76/36)	76 (15.93%)	36 (14.06%)	0.97 (0.61–1.54)	Negligible	0.8881
AB	69 (43/26)	43 (9.01%)	26 (10.16%)	1.23 (0.72–2.13)	Negligible	0.4484

* % calculated referring to N of particular characteristics in SARS-CoV-2(-) and SARS-CoV-2( +).

** Characteristics for sex were provided considering men as reference.

*** Unadjusted *p*-value.

**Table 3:** The differences in prevalence of studied symptoms between patients positive and negative for SARS-CoV-2 and the quality indices for the logistic regression based predictor for the chosen single symptoms (obtained from tenfold cross-validation).

Symptoms	N (SARS-CoV-2 (-) / SARS-CoV-2 ( +))	SARS-CoV-2 (-) Mean value ± SD	SARS-CoV-2 ( +) Mean value ± SD	OR (95% CI)	OR effect interpre-tation	OR* p*-value *	F1 (95% CI)	PPV (95% CI)	NPV (95% CI)	Diagnostic OR (95% CI)	Youden Index (95% CI)	AUC (95% CI)
Duration of symptoms (days)	1456 (975/481)	5.91 ± 10.09	4.52 ± 2.62	0.92 (0.90–0.95)	Negligible	< 0.0001	–	–	–	–	–	–
Max temperature [^o^C]	1373 (908/465)	38.05 **± **0.89	38.09 **± **0.69	1.05 (0.92–1.20)	Negligible	0.4793	–	–	–	–	–	–

Symptoms (Yes)	N (SARS–CoV-2 (-) / SARS-CoV-2 ( +))	SARS-CoV-2 (-) N (%)*	SARS-CoV-2 ( +) N (%)*	OR (95% CI)	OR effect interpre-tation	OR *p*-value **	F1 (95% CI)	PPV (95% CI)	NPV (95% CI)	Diagnostic OR (95% CI)	Youden Index (95% CI)	AUC (95% CI)
Loss of taste/smell	1388 (904/484)	206 (22.79%)	240 (49.59%)	3.33 (2.63–4.22)	Medium	< 0.0001	0.52 (0.47–0.56)	0.54 (0.5–0.58)	0.74 (0.72–0.76)	3.67 (2.49–4.85)	0.27 (0.21–0.33)	0.63 (0.60–0.67)
Dyspnea	1941 (1355/586)	521 (38.45%)	149 (25.43%)	0.55 (0.44–0.68)	Small	< 0.0001	0.47 (0.46–0.48)	0.34 (0.33–0.35)	0.78 (0.76–0.79)	1.86 (1.62–2.10)	0.13 (0.10–0.16)	0.57 (0.55–0.58)
Contact with infected person	1941 (1355/586)	315 (23.25%)	206 (35.15%)	1.79 (1.45–2.21)	Small	< 0.0001	–	–	–	–	–	–
Temperature > 38°C	1941 (1355/586)	577 (42.58%)	324 (55.29%)	1.67 (1.37–2.03)	Small	< 0.0001	0.44 (0.4–0.47)	0.36 (0.33–0.39)	0.75 (0.72–0.77)	1.79 (1.28–2.29)	0.13 (0.06–0.19)	0.56 (0.53–0.59)
GI symptoms	401 (226/175)	37 (16.37%)	8 (4.57%)	0.29 (0.14–0.60)	Medium	0.0008	0.63 (0.61–0.65)	0.47 (0.46–0.48)	0.88 (0.76–0.999)	2.38 (0.00–4.94)	0.12 (0.07–0.17)	0.56 (0.53–0.59)
Muscle aches	1394 (910/484)	534 (58.68%)	314 (64.88%)	1.30 (1.03–1.63)	Negligible	0.0242	0.47 (0.45–0.50)	0.37 (0.35–0.39)	0.69 (0.66–0.72)	1.37 (1.02–1.72)	0.06 (0.01–0.11)	0.53 (0.50–0.56)
Tiredness	419 (235/184)	49 (20.85%)	23 (12.5%)	0.54 (0.32–0.93)	Small	0.0259	0.61 (0.57–0.6)	0.46 (0.44–0.49)	0.7 (0.55–0.85)	3.04 (0.96–5.12)	0.08 (− 0.01–0.18)	0.54 (0.496–0.59)
Headache	1395 (913/482)	526 (57.61%)	305 (63.28%)	1.27 (1.01–1.59)	Negligible	0.0405	–	–	–	–	–	–
Cough	1941 (1355/586)	987 (72.84%)	452 (77.13%)	1.26 (1.00–1.58)	Negligible	0.0477	0.45 (0.43–0.47)	0.31 (0.3—0.33)	0.73 (0.70–0.77)	1.38 (0.97–1.78)	0.04 (–0.00–0.09)	0.52 (0.495–0.54)
Conjunctivitis	349 (186/163)	7 (3.76%)	0 (0.00%)	0.24 (0.05–1.14)	Medium	0.0722	0.65 (0.64–0.65)	0.48 (0.47–0.49)	1.00 (1.00–1.00)	–	0.04 (0.01–0.06)	0.52 (0.51–0.53)
Dizziness	1317 (854/463)	223 (26.11%)	103 (22.25%)	0.81 (0.62–1.06)	Negligible	0.1209	–	–	–	–	–	–
Sore throat	1390 (909/481)	402 (44.22%)	194 (40.33%)	0.85 (0.68–1.07)	Negligible	0.1633	–	–	–	–	–	–
Loss of appetite	360 (193/167)	3 (1.55%)	5 (2.99%)	1.65 (0.51–5.33)	Small	0.4052	0.05 (0.00–0.11)	0.71 (0.16–1.00)	0.54 (0.53–0.55)	0.88 (0.00–4.82)	0.01 (–0.02–0.05)	0.51 (0.49–0.52)
Skin reactions	1374 (894/480)	34 (3.80%)	19 (3.96%)	1.08 (0.63–1.88)	Negligible	0.7731	–	–	–	–	–	–

* % calculated referring to N of particular characteristics in SARS-CoV-2(-) and SARS-CoV-2( +).

** Unadjusted p-value.

### Symptoms and diseases differentiating COVID-19 from other infections

**Figure 1:**
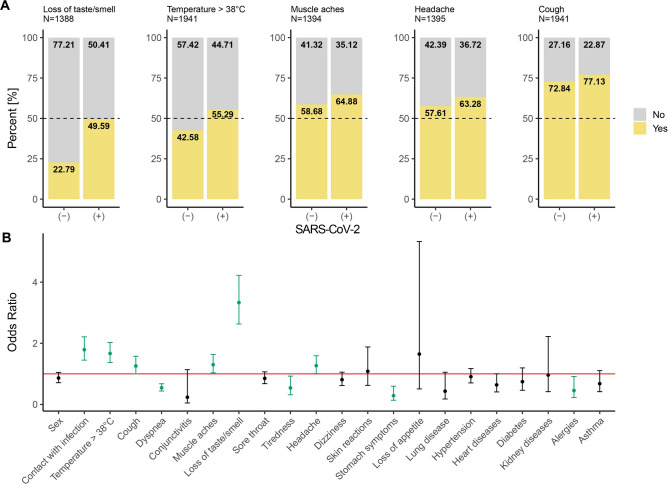
Results of statistical analysis. **Panel A** stacked bar plots showing frequencies of symptoms regarding COVID-19 diagnosis. Symptoms having a higher odds ratio in SARS-CoV-2 infected patients were shown. **Panel B** odds ratio for all symptoms and coexisting diseases with 95% confidence intervals. The red line shows the level of odds ratio equal to 1. Significant odds ratios were coloured green.

Next, we focused on a list of symptoms (Table 3) and coexisting diseases (Table 2) to identify the ones that have a significant association with COVID-19. The strongest co-occurrence was observed between loss of taste or smell and COVID-19 disease (Fig. 1, Panel A).

The odds of getting a positive COVID-19 diagnosis are more than three times bigger among people with loss of taste or smell (Fig. 1, Panel B). The observed OR value corrected after the subgroup size imbalance interprets as the medium effect. Other symptoms coexisting with early SARS-CoV-2 infection were high temperature (> 38°C), muscle aches, headache and cough, although, for all, the effect is small or negligible. On the other hand, a negative association with COVID-19 was observed for dyspnea, gastrointestinal symptoms (GI symptoms) and tiredness. These symptoms were less frequent in the group of patients with early SARS-CoV-2 infection. The observed effects were medium level at most as the odds of getting a positive COVID-19 diagnosis were about two times lower for patients observing symptoms mentioned above (Fig. 1, Panel B). Notably, a negative relation with SARS-CoV-2 infection was also observed for the allergies with a small effect.

Table 3 also presents the summary of the chosen quality indices for the single symptom logistic regression (LR) based predictor of SARS-CoV-2 status. The mean value and its 95% confidence interval obtained in tenfold cross-validation are provided there. The mean AUC (Area Under the Curve) varies from 50.7% (loss of appetite) to 63.4% (loss of taste/smell). The maximum negative predictive value (NPV), equal to 87.7%, was observed for GI symptoms with a positive predictive value (PPV) at the level of 47.0%.

### Pairwise association of symptoms

**Figure 2:**
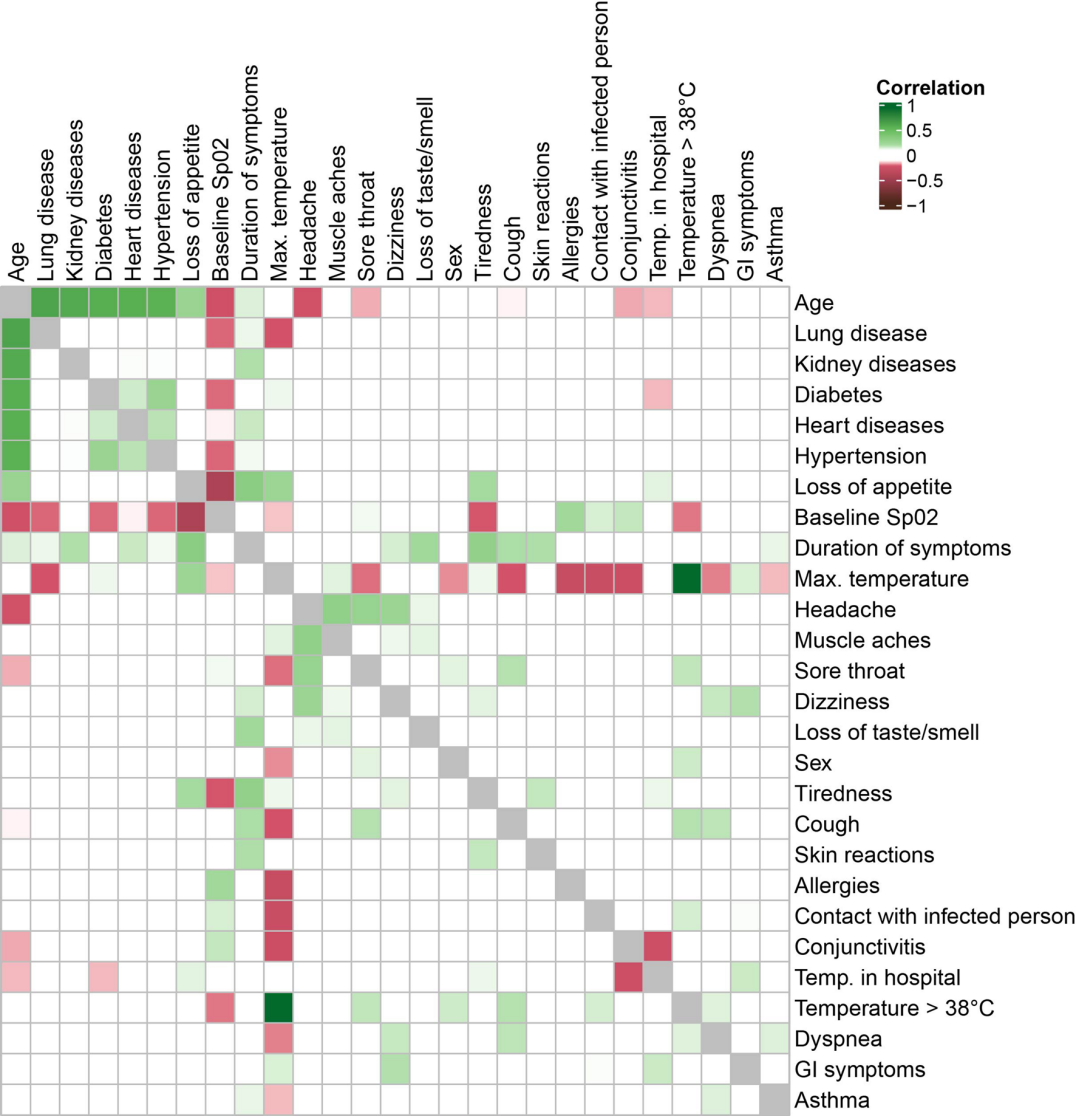
Heatmap with correlation coefficients for pairwise comparison of queries. Blood type was ignored due to different interpretation of the effect size coefficient. Comparisons with at least a small effect size have been coloured, i.e. values of correlation within the range (− 0.1, 0.1) were left blank.

Subsequently, we examined the correlation between every pair of surveyed queries. The highest correlations were observed between age and coexisting diseases: lung disease, kidney disease, diabetes, heart disease and hypertension (Fig. 2), which is in agreement with the general observations. A medium-level association was observed between loss of appetite and saturation (i.e. baseline Sp02; negative association) and duration of symptoms (positive association). Loss of appetite also coincides with patient age and maximum temperature with a small effect. While considering the headache, it co-occurs with a series of symptoms: muscle aches, sore throat and dizziness with positive small but very close to medium effect, with sex and loss of taste or smell with small positive effect, and with age with the small negative association. Another positive association (with small or close to medium effect) were observed between duration of symptoms and tiredness, loss of appetite, loss of taste or smell, cough and skin reactions. Interestingly, maximum temperature correlates negatively with a list of characteristics: allergies, contact with infection and conjunctivitis, with small but close to medium effect. Moreover, it correlates negatively with cough, lung disease, sore throat, dyspnea, and saturation with a small effect. Similarly, a negative correlation was observed between saturation and age, tiredness, and most of the coexisting diseases (with small effect).

### Occurrence and co-occurrence of symptoms

**Figure 3:**
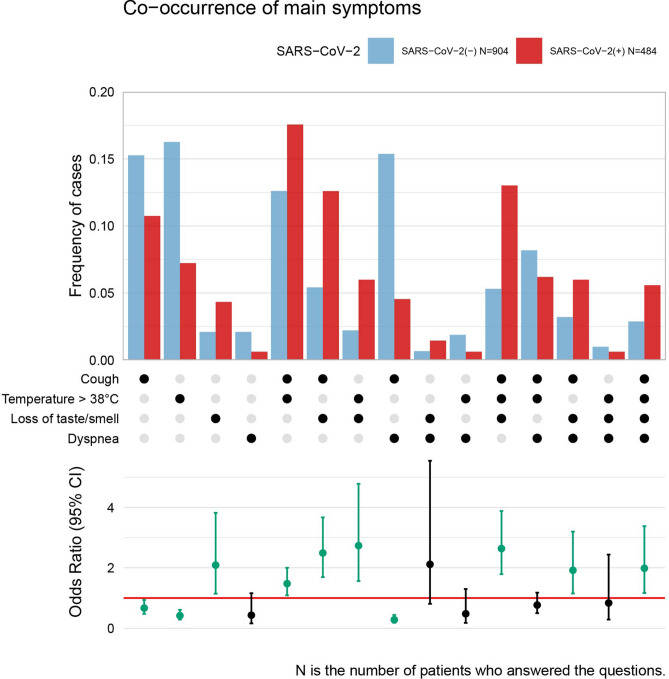
Frequency of patients observing one symptom or a combination of symptoms regarding the total number of patients from a certain group (infected or not infected with SARS-CoV-2) who answered all four analysed queries. Dots in the middle part indicate which symptom or combination of symptoms the bars in the upper part of the plot refer to. For example, about 15% of patients with negative COVID-19 diagnosis reported only cough (and no high temperature, no dyspnea, no loss of taste/smell), whereas, from the group of patients with a positive diagnosis, about 11% of patients reported only cough (no other symptoms from the list). The lower part of the plot shows the odds ratio with 95% confidence intervals for comparison of SARS-CoV-2 diagnosis with a corresponding symptom or symptom combination. The red line shows the level of odds ratio equal to 1. Significant odds ratios (*p* < 0.05) were coloured green.

Next, we examined if the co-occurrence of symptoms is specific for COVID-19 diagnosis. Table 4 and Fig. 3 shows the frequency of patients observing one symptom only (and no other signs from the list) or a particular combination of symptoms from a list of four queries: temperature > 38℃, cough, dyspnea, and loss of taste or smell. Table 4 also presents the value of the prediction quality indices for the occurrence and co-occurrence of the analysed symptoms derived from the whole data set. The highest effect was observed for the co-occurrence of two symptoms only: cough and dyspnea, with no temperature > 38℃ and no loss of taste or smell, which seemed protective against COVID-19 and was about 3.5 times more frequent among people with negative COVID-19 diagnosis (*p* < 0.0001, NPV = 86%, PPV = 38%, DOR = 3.82). Observation of only high temperature or only cough speaks against COVID-19 too (OR = 0.42 and 0.67 respectively, *p* < 0.0001 and *p* = 0.0201). The COVID-19 prediction indices for such situations are NPV = 81%, PPV = 37% and DOR = 2.49 for the observation of high temperature (> 38℃) only, and NPV = 73%, PPV = 36% and DOR = 1.50 for cough only, respectively.

**Table 4:** The differences in occurrence and co-occurrence of chosen symptoms between patients positive and negative for SARS-CoV-2 and the quality indices for the logistic regression based predictor (derived from all patients).

Symptoms and combinations of symptoms	N (SARS-CoV-2 (-) / SARS-CoV-2 ( +))	SARS-CoV-2 (-) N (%)*	SARS-CoV-2 ( +) N (%)*	OR (95% CI)	OR effect interpretation	OR p-value **	F1	PPV	NPV	Diagnostic OR
Temperature > 38°C (protective)	1388 (904/484)	147 (16.26%)	35 (7.23%)	0.42 (0.29–0.61)	Small	< 0.0001	0.53	0.37	0.81	2.49
Cough (protective)	1388 (904/484)	138 (15.27%)	52 (10.74%)	0.67 (0.48–0.94)	Small	0.0201	0.51	0.36	0.73	1.50
Dyspnea (none)	1388 (904/484)	19 (2.10%)	3 (0.62%)	0.43 (0.16–1.16)	Small	0.0960	–	–	–	–
Loss of taste/smell (risk)	1388 (904/484)	19 (2.10%)	21 (4.34%)	2.09 (1.14–3.82)	Small	0.0166	0.08	0.53	0.66	2.11
Temperature > 38°C & Cough (risk)	1388 (904/484)	114 (12.61%)	85 (17.56%)	1.48 (1.09–2.00)	Small	0.0125	0.25	0.43	0.66	1.48
Temperature > 38°C & Dyspnea (none)	1388 (904/484)	17 (1.88%)	3 (0.62%)	0.48 (0.18–1.30)	Small	0.1480	–	–	–	–
Temperature > 38°C & Loss of taste/smell (risk)	1388 (904/484)	20 (2.21%)	29 (5.99%)	2.73 (1.56–4.78)	Small	0.0004	0.11	0.59	0.66	2.82
Cough & Dyspnea (protective)	1388 (904/484)	139 (15.38%)	22 (4.55%)	0.28 (0.18–0.44)	Moderate	< 0.0001	0.54	0.38	0.86	3.82
Cough & Loss of taste/smell (risk)	1388 (904/484)	49 (5.42%)	61 (12.60%)	2.49 (1.69–3.67)	Small	< 0.0001	0.21	0.55	0.67	2.52
Dyspnea & Loss of taste/smell (none)	1388 (904/484)	6 (0.66%)	7 (1.45%)	2.12 (0.81–5.55)	Small	0.1272	–	–	–	–
Temperature > 38°C & Cough & Dyspnea (none)	1388 (904/484)	74 (8.19%)	30 (6.20%)	0.77 (0.50–1.18)	Negligible	0.2259	–	–	–	–
Temperature > 38°C & Cough & Loss of taste/smell (risk)	1388 (904/484)	48 (5.31%)	63 (13.02%)	2.64 (1.79–3.88)	Small	< 0.0001	0.21	0.57	0.67	2.67
Temperature > 38°C & Dyspnea & Loss of taste/smell (none)	1388 (904/484)	9 (1.00%)	3 (0.62%)	0.84 (0.29–2.44)	Negligible	0.7442	–	–	–	–
Cough & Dyspnea & Loss of taste/smell (risk)	1388 (904/484)	29 (3.21%)	29 (5.99%)	1.92 (1.15–3.20)	Small	0.0124	0.11	0.50	0.66	1.92
Temperature > 38°C & Cough & Dyspnea & Loss of taste/smell (risk)	1388 (904/484)	26 (2.88%)	27 (5.58%)	1.99 (1.17–3.38)	Small	0.0115	0.10	0.51	0.66	2.00

* % calculated referring to N of particular characteristics in SARS-CoV-2(-) ad SARS-CoV-2( +).

** Unadjusted *p*-value.

Among the single symptom occurrence, only loss of taste/smell brings a significant increase of COVID-19 odds with OR = 2.09 and *p* = 0.0166, and NPV = 66%, PPV = 53% and DOR = 2.11. The co-occurrence of cough and loss of taste/smell without high temperature and dyspnea increases slightly the risk of SARS-CoV-2 infection (*p* < 0.0001, OR = 2.49, NPV = 67%, PPV = 55%, DOR = 2.52). Observing the co-occurrence of the third symptom: high temperature or even presence of all four symptoms, do not significantly increase the odds (OR = 2.67 and OR = 2.00, respectively). Adding four more symptoms to the co-occurrence analysis presented a more complex picture of the associations with COVID-19 disease (see Supplementary Figure S2). Muscle aches, headache, sore throat and dizziness have been inspected for co-appearance with symptoms mentioned above.

Occurrence of muscle aches or headache only gives nonsignificant OR for COVID-19 diagnosis, while combination with some of the main symptoms changes OR significantly. For example, the co-occurrence of headache with cough and loss of taste/smell increases the estimate of odds ratio from OR = 2.49 to OR = 3.74, while co-occurrence of muscle ache with the two main symptoms does not impact their OR (2.49 versus 2.15). However, the co-occurrence of the four mentioned above symptoms (cough, loss of smell/taste, headache and muscle ache) results in higher OR = 4.72.

### The prediction power of the symptom-based classifiers

Additionally, to support the early-stage symptom-based decision on the patient being or not SARS-CoV-2 positive, the LR-based predictors were also built for a different subset of the nine main symptoms considered (N = 398). The summary of the results is presented in Supplementary Table S2 which provides the expanded list of the classification quality indices and their 95% CI obtained in tenfold cross-validation. Among the single-symptom models, the loss of taste/smell-based classification gives the best AUC = 0.634 (95% CI 0.603–0.665) and DOR = 3.670 (95% CI 2.494–4.847). The highest AUC (> 70%) and DOR (> 3) values were obtained for the three symptom signatures: (1) temperature > 38°C & cough & loss of taste/smell & GI symptoms (AUC 0.704, 95% CI: 0.632–0.776 and DOR 8.991, 95% CI: 3.792–14.191); (2) dyspnea & cough & loss of taste/smell & GI symptoms (AUC 0.705, 95% CI: 0.640–0.769, DOR 5.566, 95% CI: 2.806–8.327) and (3) cough & muscle aches & loss of taste/smell & GI symptoms (AUC 0.711, 95% CI: 0.647–0.776, DOR 5.738, 95% CI: 3.045–8.430). The correction after the age and sex has not changed the above estimates significantly.

### Profiles of patients infected with SARS-CoV-2 regarding sex and age subsets

Finally, considering hormonal and immune response differences between men and women, as well as between younger and older people, we performed separate analyses for the mentioned subsets. The age threshold was set to 46 years as indicated by the Gaussian Mixture Model (GMM) decomposition of the age characteristic^28^.

#### Sex subsets

Analysis of association between COVID-19 diagnosis and a single query showed that both subsets are characterised by associations between COVID-19 diagnosis and contact with an infected person, temperature > 38℃, dyspnea, and loss of taste or smell. Cough, muscle aches, and headache appear significantly more frequently only in SARS-CoV-2 infected men, whereas GI symptoms are less frequent only in men tested positive. The odds for women are not significant (see Supplementary Table S3). On the other hand, decreased odds of sore throat and heart disease are significant exclusively in women. Notably, the correlation between lung disease and saturation is observed only in men and not in women (see Supplementary Figure S3, Panel a). Analysis of co-occurrence of four main symptoms with COVID-19 diagnosis showed a similar pattern for men and women. However, among a subset of patients who answered all questions about temperature > 38℃, cough, dyspnea and loss of taste or smell, women have got a significantly higher odds of positive COVID-19 diagnosis when observing the loss of taste or smell only (*p* = 0.0177, OR = 2.61, Supplementary Figure S4or all four symptoms (*p* = 0.0276, OR = 2.23). Limiting the analysis to a subset of patients infected with SARS-CoV-2 (see Supplementary Figure S5, Panel a), the biggest percentage of men observed a combination of temperature > 38℃ and cough (20%), whereas women most frequently observe co-occurrence of loss of taste or smell and cough (17%).

#### Age subsets

Only in the younger subset, we have an association between COVID-19 diagnosis and sex, where odds of being infected with SARS-CoV-2 are about 0.7 times lower in women than in men (see Supplementary Table S3). Both subsets are characterised by the coincidence of COVID-19 diagnosis and contact with the infection, temperature > 38℃, dyspnea and loss of taste or smell. In addition, younger patients have a positive association between COVID-19 diagnosis and i) headache and ii) muscle aches, where odds of being infected with SARS CoV-2 are about 1.5 times bigger while having these symptoms. Younger people also show negative associations between positive COVID-19 diagnosis and sore throat, dizziness, GI symptoms, and asthma. On the other hand, older people infected with SARS-CoV-2 observe cough more often than not infected people and have heart diseases less often. Analysis of pairwise correlation between all queries showed a bigger number of at least small correlations for older patients. Not only do we observe a correlation between coexisting diseases, but also a negative correlation between age and a list of symptoms, including saturation, cough, headache or skin reactions (see Supplementary Figure S3, Panel b).

**Figure 4:**
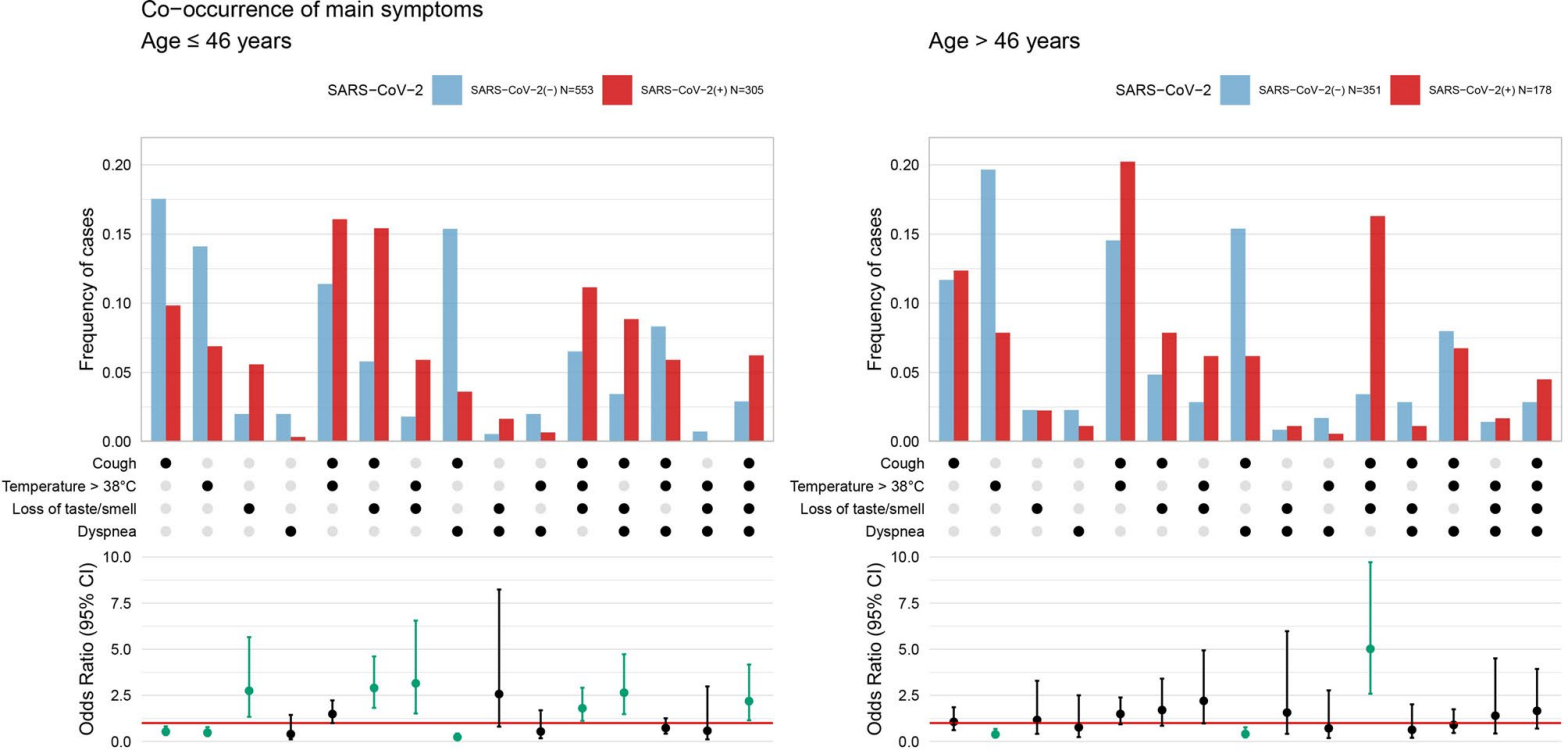
Frequency of patients observing one symptom or a combination of symptoms regarding the total number of patients from a certain group (infected or not infected with SARS-CoV-2) who answered all four analysed queries, considered in two age subsets. Dots in the middle part indicate which symptom or combination of symptoms the bars in the upper part of the plot refer to. The lower part of the plot shows an odds ratio with 95% confidence intervals for comparison of SARS- CoV-2 diagnosis with a corresponding symptom or symptoms combination. The red line shows the level of odds ratio equal to 1. Significant odds ratios were coloured green.

Co-occurrence of four main symptoms with COVID-19 diagnosis analysis showed that most of the associations are significant for younger people and not for older ones (Fig. 4). From the subset of people who answered all questions about temperature > 38℃, cough, dyspnea and loss of taste or smell, the following symptoms were associated with COVID-19 diagnosis among older patients: (1) cough and dyspnea (as a protective factor), (2) temperature > 38℃, cough and loss of taste or smell (risk factor) and (3) high temperature (above 38℃) only, with no other symptoms (serves as the protective factor against SARS-CoV-2 infection). Younger people had the same relations as the entire population, with an exception to temperature > 38℃ and cough exhibiting lack of association. Considering analysis only on a subset of patients infected with SARS-CoV-2 (see Supplementary Figure S5, Panel b), the biggest percentage of younger patients observed a combination of temperature > 38℃ and cough, as well as the co-occurrence of loss of taste or smell and cough (about 16% both combinations). Older people more frequently observe co-occurrence of temperature > 38℃ and cough (21%), as well as the co-occurrence of loss of taste or smell, cough and high temperature (17%).

## Discussion

The main findings regarding the analysis of the single symptoms are in compliance with the already published reports^29–34^. The main importance of this study is the frequency and correlation analyses among the symptoms and clinical characteristics of SARS-CoV-2 infected versus control group consisting of people with suspected SARS-CoV-2 infection, which was not confirmed through PCR tests of material from upper respiratory system swabs.

This approach enables not only characterising the COVID-19 symptomatology but also a determination of prognostic significance for each symptom in COVID-19 diagnostics. Moreover, dependencies between symptoms and their interactions have been determined, giving a comprehensive view of the disease. For instance, in the studied population, dyspnea as a symptom, often indicated as crucial in SARS-CoV-2 diagnostics^35,36^, occurred more frequently in non-infected individuals. This fact may suggest the subjectivity of the symptom and possible connection to stress resulting from fear of SARS-CoV-2 infection or other underlying diseases. Alternatively, it may signal that dyspnea and fatigue (also less frequent among SARS-CoV-2 infected) may develop at later disease stages^37^. Noteworthy, the occurrence of cough and dyspnea without fever or smell and taste impairment significantly diminishes the risk of testing SARS-CoV-2 positive, contrary to patients with acute lung disease or atypical lung inflammation. A similar situation occurs in gastrointestinal symptoms, which are associated with SARS-CoV-2 symptomatology; however, their appearance was much rarer compared to other infections that may have been treated incorrectly as COVID-19 symptoms. Consequently, the occurrence of GI symptoms alone presented little prognostic value.

Remarkably, we were able to distinguish certain sets of symptoms based on correlation analysis which form a logical entity and discriminate their pathogenic association. For instance, the headache was linked with muscle pain and loss of taste and smell, forming a neurological panel^38^ which correlated negatively with age. Furthermore, loss of appetite was observed to a greater degree in older patients with longer disease duration and higher temperature. Nonetheless, the higher temperature was found in men and people who pointed out having neither asthma, allergies, nor dyspnea. It might indicate that the following population is more susceptible to anergy.

The observed in our study, positive association between baseline SpO2 and allergies, mainly respiratory allergies, is currently a very important topic. It is well known that innate antiviral responses are decreased but also an expression of SARS-Cov2 receptor ACE2 upregulated in epithelial cells is chronic rhinosinusitis^39,40^ which could aggravate the course of COVID-19. On the other hand, majority of those patients receive different medications modifying the course of disease, which include inhaled corticosteroids, short- or long-acting β agonist. Interestingly, recent data suggest that inhaled corticosteroids could decrease ACE2 expression^40^ but, even more importantly, reduced the likelihood of needing urgent medical in the early stages of COVID-19 as shown in STOIC randomised controlled trial^41^. This association undoubtedly calls for further investigations.

The dependency analysis and co-occurrence of symptoms showed that in the entire group of single symptoms, loss of taste and smell proved to be the most strongly discriminating. Additionally, in the case of symptom interactions, combining loss of taste and smell with temperature > 38°C and cough does not increase the odds ratio significantly but shrinks its confidence interval range, therefore, recognition precision.

Further, interesting associations were discovered in groups of men and women, e.g., the significance of headache in men. It may be attributed to more frequent migraines and tension headaches in women who do not take notice of such pains^42^. Moreover, age appears to be a significant factor—the symptomatology changes around the age of 46. It is clearly visible that in younger people, only taste or smell impairment is sufficient, whereas, in older patients, for similar measurements to be observed, there is a need for the co-occurrence of fever and cough.

It is of significance to note that the findings in this work are based on data collected in a single hospital centre, and therefore, depending on the demographic profile, some variation may be observed between regions. Another limitation of the study may be attributed to the lack of follow-up surveys which could enhance the understanding of early stage symptoms’ impact. It also leads to consider the absence of information on mortality rates in this specific group of patients that inhibits from profiting fully from information regarding, e.g. comorbidities.

The presented results not only objectify the significance of single symptoms and their interactions in COVID-19 diagnostics but also demonstrate diverse symptomatology in particular groups of individuals, e.g. younger versus older, men versus women. The essential observation may be concluded through the fact that clearly in younger people, loss of taste and smell is a sufficient symptom for SARS-CoV-2 infection when in older patients, the symptom needs to co-occur with fever and cough. The results of this study may prove beneficial in the process of patient selection for PCR or antigen testing towards SARS-CoV-2 infection by means of e.g., remote clinical services, especially in the case where testing the entire population is infeasible, or web-based self-assessment tools.

## Conclusions

Following analysis showed that patients infected with SARS-CoV-2 more frequently observe the loss of taste or smell, high temperature > 38℃ and cough. Notably, dyspnea reported by other research groups as more frequent among people with positive COVID-19 diagnosis here was less common among patients infected with SARS-CoV-2 at an early disease stage. Similarly, co-occurrence of cough and dyspnea appeared with lower rates in people with positive COVID-19 diagnosis. On the other hand, co-occurrence of loss of taste or smell with a list of symptoms was more frequent among people with positive COVID-19 diagnosis, including combinations with (1) temperature > 38℃, (2) cough, or (3) temperature > 38℃ and cough. Noteworthy, different responses are observed by people younger and older than 46 years. Older patients infected with SARS-CoV-2 more often observe cough and its co-occurrence with high temperature and loss of taste or smell. Younger people with positive COVID-19 diagnosis frequently report headaches and muscle aches, in addition to the main symptoms of loss of taste or smell, high temperature > 38℃ and cough. Worth noticing that in younger people, only taste or smell impairment is sufficient, whereas, in older patients, for similar measurements to be observed, there is a need for the co-occurrence of fever and cough.

## Methods

### Data acquisition

Data were collected in the course of DECODE project (Data drivEn COVID-19 DEtection)^43^ from paper surveys presented to the patients of the Department of Infectious Diseases and Hepatology, Bytom, Poland who were tested for SARS-CoV-2 infection. Patients were tested due to the presence of any symptoms of respiratory tract infection and/or direct contact with SARS-CoV-2 infected subjects (asymptomatic group). In all subjects included vital signs, complete physical examination and oxygen saturation prior to O_2_ supplementation were performed. SARS-CoV-2 infection was determined by use of RT-PCR from nasopharyngeal swabs with 2019-nCOV Triplex RT-qPCR Detection Kit, Vazyme with a lower limit of detection 200 copies of RNA/mL. The study was approved by Bioethics Committee of Silesian Medical Chamber in Katowice, Poland. Patients provided written informed consent to participate in the study. We complied with all relevant ethical regulations and guidelines. Surveys were collected in the time range from March 3rd to September 30th, 2020. During the whole time of data acquisition, every patient who observed any symptom or had contact with an infected person was allowed to participate in the study. Patients reported their health state at the early stage of the disease. Captured data consist of demographics, previous contacts with SARS-CoV-2 infection, current symptoms, medical history including co-medications and vital signs with oxygen saturation. The collection of surveys was not supervised, thus resulting in a number of missing information. Every survey was completely anonymised to prevent personal data leak. Next, a database was created, including information from all survey queries.

### Comparative analysis

Statistical analysis was applied to determine queries having different characteristics between patients infected and not infected with SARS-CoV-2. The odds ratio (further denoted as OR) was estimated and accompanied by its 95% confidence interval (CI) as well as test for OR difference to one^44^. For categorical variables (i.e. blood type and all discrete having presence/absence state), Wilson frequency estimator was used^45^ in the case of frequencies < 10%. For continuous variables, OR was computed from logistic regression coefficients. The significance level for statistical testing was set to 0.05. Moreover, the odds ratios served as a measure of effect size.

### Correlation analysis

To find associations between the analysed features, the following effect size coefficients were calculated: (1) Spearman’s correlation coefficient between two continuous variables (further denoted as R), (2) Cramer’s V coefficient (further denoted as V) between two discrete variables^46^, and (3) Wendt’s biserial coefficient of correlation (further denoted as RBCC) between continuous and discrete variable^47^. All pairwise comparisons were examined. For the unbiased estimation of V coefficient, χ2 statistics for the independence test was computed with Yates continuity correction^48^ in the case of low occurrence counts (n < 10).

### Effect size interpretation

The effect size quantification was done for absolute values of relevant statistics. Interpretation of OR is defined as small, medium and large for the relative thresholds considering allocation ratio for every query independently^49^. Interpretation of effect size for R, V and RBCC coefficients is the same and is defined as small, medium and large for the following thresholds: 0.1, 0.3 and 0.5, respectively^50^.

### SARS-CoV-2 status predictor construction and evaluation

The logistic regression models for single characteristic and for the combinations of co-occurring symptoms were built. Patients who failed to provide the necessary information in terms of particular characteristics used for a model construction were excluded from the training process. Each model aims to classify a patient as SARS-CoV-2 ( +) or (−) based on the set of input features. The models were evaluated with a tenfold cross-validation procedure. All available symptomatic samples were split into 10 subsets with preserved SARS-CoV-2 ( +)/(−) distribution. Youden index maximisation served for the selection of probability cut-off value from the range 0.1–0.9. Mean values and their 95% confidence intervals of performance measures are provided.

## Supplementary Information

Supplementary Information 1.

Supplementary Information 2.

## Data Availability

The datasets generated during and/or analysed during the current study are available from the corresponding author on request.
