# Supplementary material for: Symptom-based early-stage differentiation between SARS-CoV-2 versus other respiratory tract infections—Upper Silesia pilot study
Source: Sci Rep. 2021 Jun 30;11:13580. doi: 10.1038/s41598-021-93046-6 (PMC8245528; doi:10.1038/s41598-021-93046-6)
Supplement: Supplementary file 2 — Supplementary Information 2. [file 41598_2021_93046_MOESM2_ESM.pdf]

SUPPLEMENTARY MATERIALS

Symptom based early stage differentiation between SARS-CoV-2 versus other respiratory tract infections - Upper Silesia pilot study

Justyna Mika[1], Joanna Tobiasz[1], Joanna Zyla[1], Anna Papiez[1], Małgorzata Bach[2], Aleksandra Werner[2], Michał Kozielski[3], Mateusz Kania[4], Aleksandra Gruca[3], Damian Piotrowski[5], Barbara Sobala-Szczygieł[5], Bożena Włostowska[5], Paweł Foszner[4], Marek Sikora[3], Joanna Polanska[1]*, Jerzy Jaroszewicz[5]

[1]Department of Data Science and Engineering, Silesian University of Technology, Gliwice, Poland,

[2]Department of Applied Informatics, Silesian University of Technology, Gliwice, Poland,

[3]Department of Computer Networks and Systems, Silesian University of Technology, Gliwice, Poland,

[4]Department of Graphics, Computer Vision and Digital Systems, Silesian University of Technology, Gliwice, Poland,

[5]Department of Infectious Diseases and Hepatology, Medical University of Silesia, Katowice, Poland

*corresponding author: joanna.polanska@polsl.pl

Supplementary figure legends

Fig. S1. **The distribution of missing values across sample**.

Fig. S2. **Frequency of patients observing one symptom or a combination of symptoms regarding the total number of patients from a certain group (infected or not infected with SARS-CoV-2) who answered all eight analysed queries**. Dots in the lower part indicate which symptom or a list of symptoms is shown by the bars in the upper part of the plot. For example: almost 4% of patients with negative COVID-19 diagnosis reported only high temperature (and not cough, muscle aches, headache, sore throat, loss of taste/smell, dyspnea or dizziness), whereas from the group of patients with positive diagnosis, about 1% of patients reported only high temperature. Significant odds ratio were coloured green. Combinations of symptoms with frequencies < 0.01 were not shown here.

Fig. S3. Heatmaps with correlation coefficients for pairwise comparison of queries. Blood type was ignored due to different interpretation of effect size coefficient. Comparisons with at least small effect size have been coloured, i.e. values of correlation within the range (-0.1, 0.1) were not coloured. Panel a shows heatmaps for men and women. Panel b shows heatmaps for two age subsets (younger and older than 46 years).

Fig. S4. Frequency of patients observing one symptom or a combination of symptoms regarding the total number of patients from a certain group (infected or not infected with SARS-CoV-2) who answered all four analysed queries, considered men and women separately. Dots in the middle part indicate which symptom or combination of symptoms the bars in the upper part of the plot refer to. The lower part of the plot shows odds ratio with 95% confidence intervals for comparison of SARS-CoV-2 diagnosis with a corresponding symptom or symptoms combination. The red line shows the level of odds ratio equal to 1. Significant odds ratio were coloured green. The odds ratio is significant if the confidence interval does not include the value of 1 (red line).

Fig. S5. **Venn diagrams showing co-occurrence of four main symptoms among people infected with SARS-CoV-2**. Colour scale shows the frequency of symptoms ranging from small frequency (white) to high frequency (red). Panel a – Venn diagrams for men and women separately. Panel b – Venn diagrams for younger and older subsets of patients.

Supplementary tables:

Table S1. The detailed characterisation of the sample.

Table S2. The quality indices for logistic-regression-based predictors of the early-stage SARS-CoV-2 infection for the chosen subsets of the nine main symptoms (10-fold cross-validation).

Table S3. The sex- and age-dependent prevalence of studied symptoms among patients positive and negative for SARS-CoV-2.

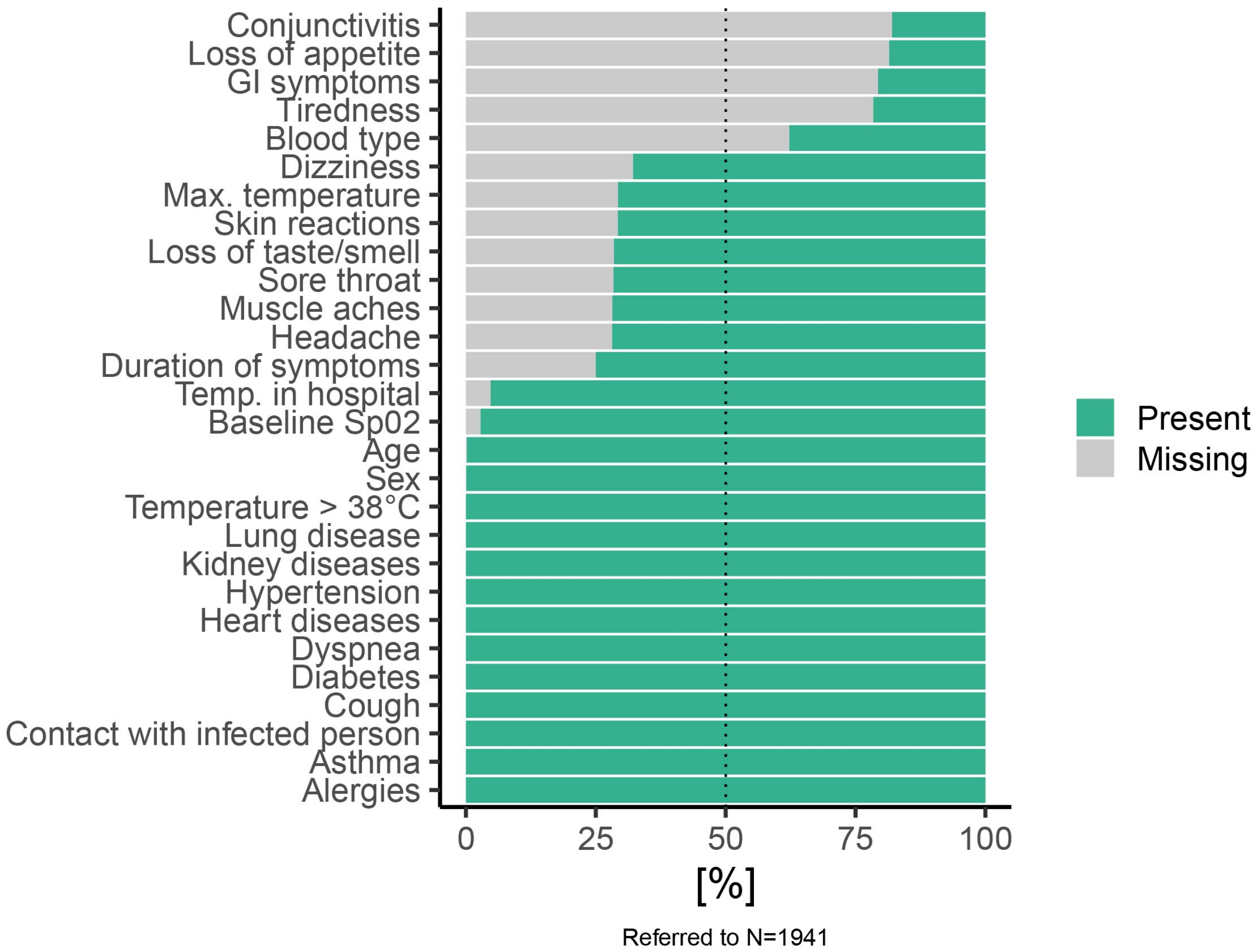

Conjunctivitis
Loss of appetite
GI symptoms
Tiredness
Blood type
Dizziness
Max. temperature
Skin reactions
Loss of taste/smell
Sore throat
Muscle aches
Headache
Duration of symptoms
Temp. in hospital
Baseline Sp02
Age
Sex
Temperature > 38°C
Lung disease
Kidney diseases
Hypertension
Heart diseases
Dyspnea
Diabetes
Cough
Contact with infected person
Asthma
Alergies
0
25
50
75
100
[%]
Present
Missing
Referred to N=1941

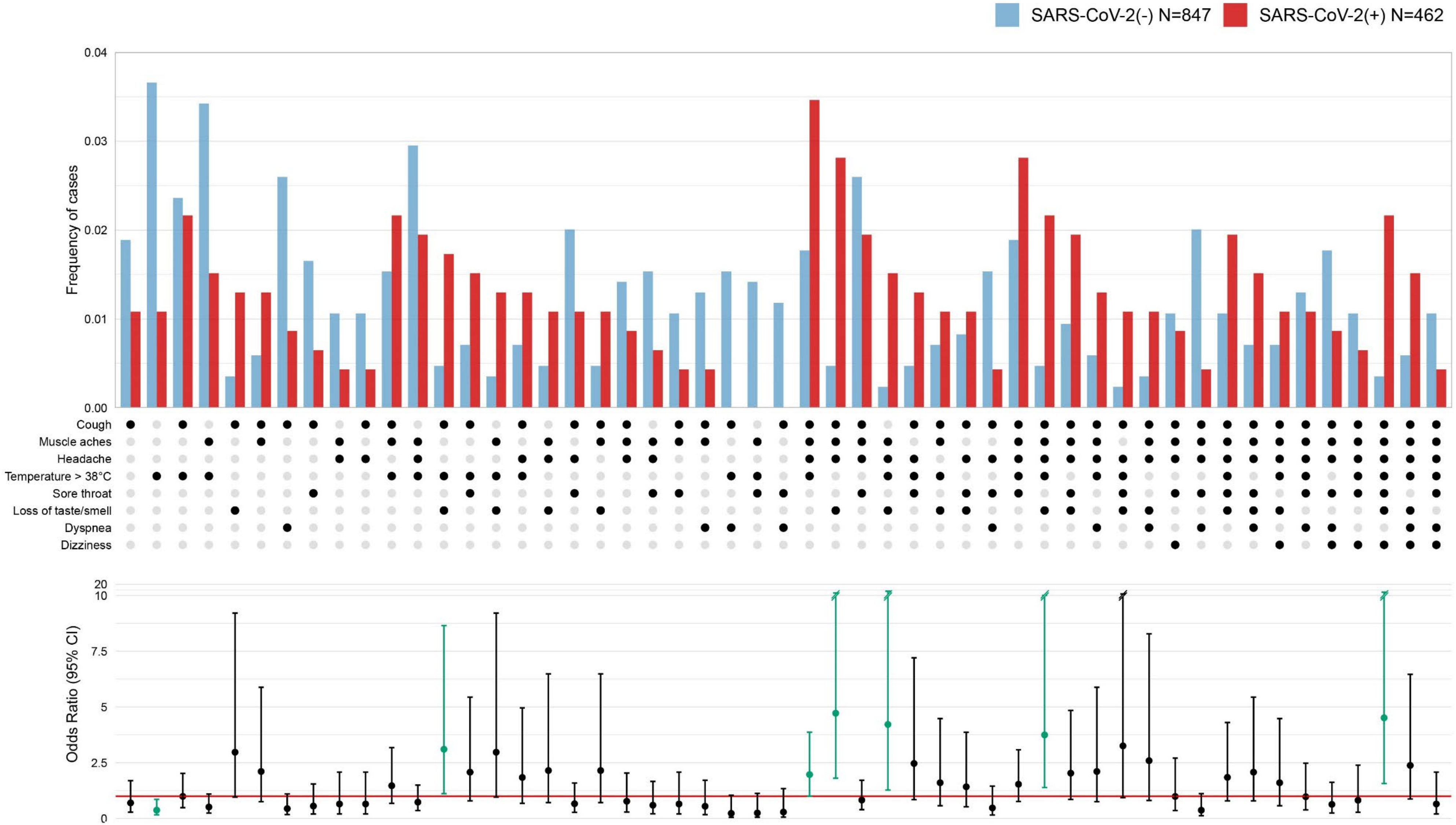

N is the number of patients who answered the questions.

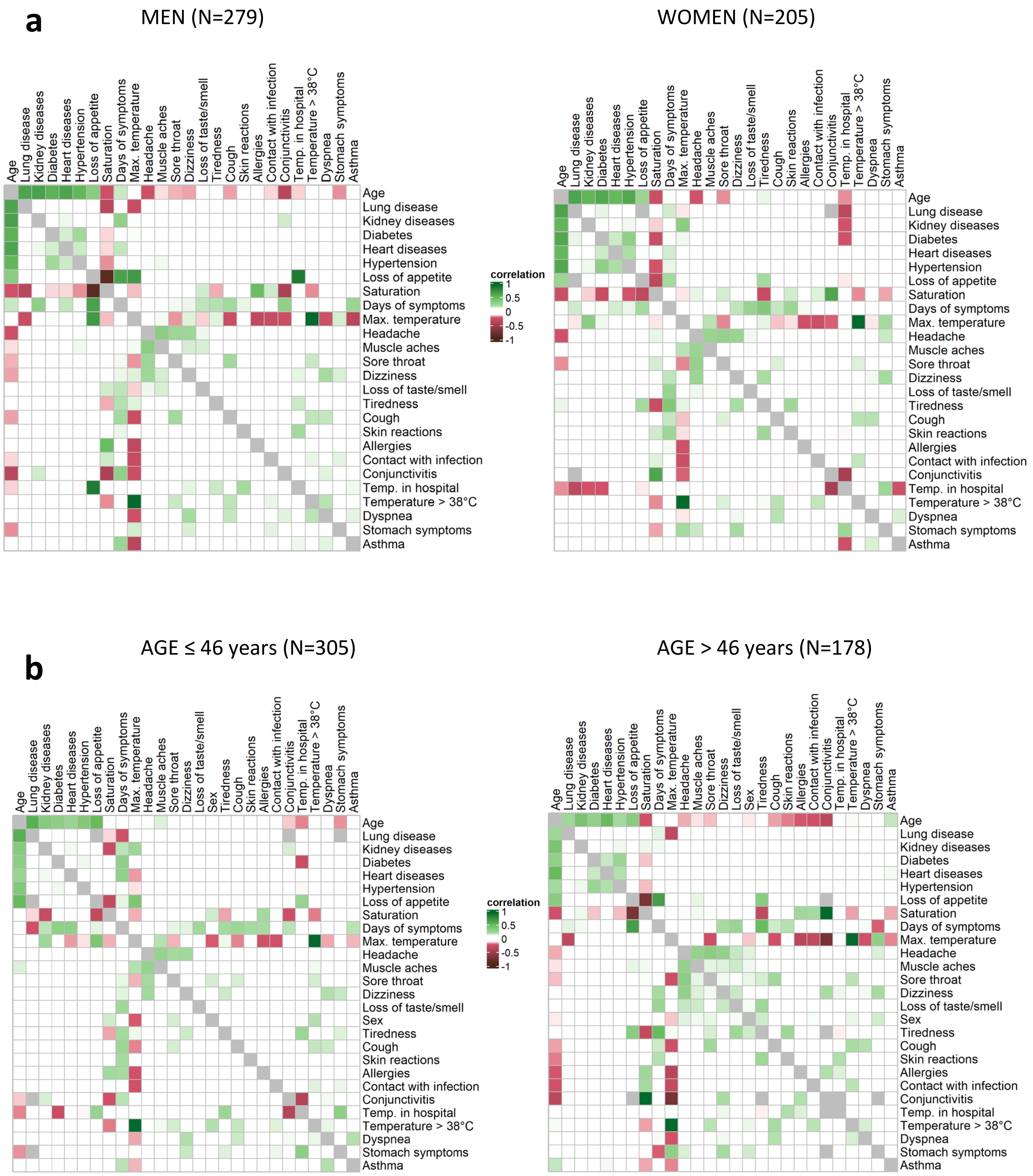
a
MEN (N=279)
WOMEN (N=205)
b
AGE ≤ 46 years (N=305)
AGE > 46 years (N=178)
correlation
1
0.5
0
-0.5
-1
Age
Lung disease
Kidney diseases
Diabetes
Heart diseases
Hypertension
Loss of appetite
Saturation
Days of symptoms
Max. temperature
Headache
Muscle aches
Sore throat
Dizziness
Loss of taste/smell
Sex
Tiredness
Cough
Skin reactions
Allergies
Contact with infection
Conjunctivitis
Temp. in hospital
Temperature > 38°C
Dyspnea
Stomach symptoms
Asthma

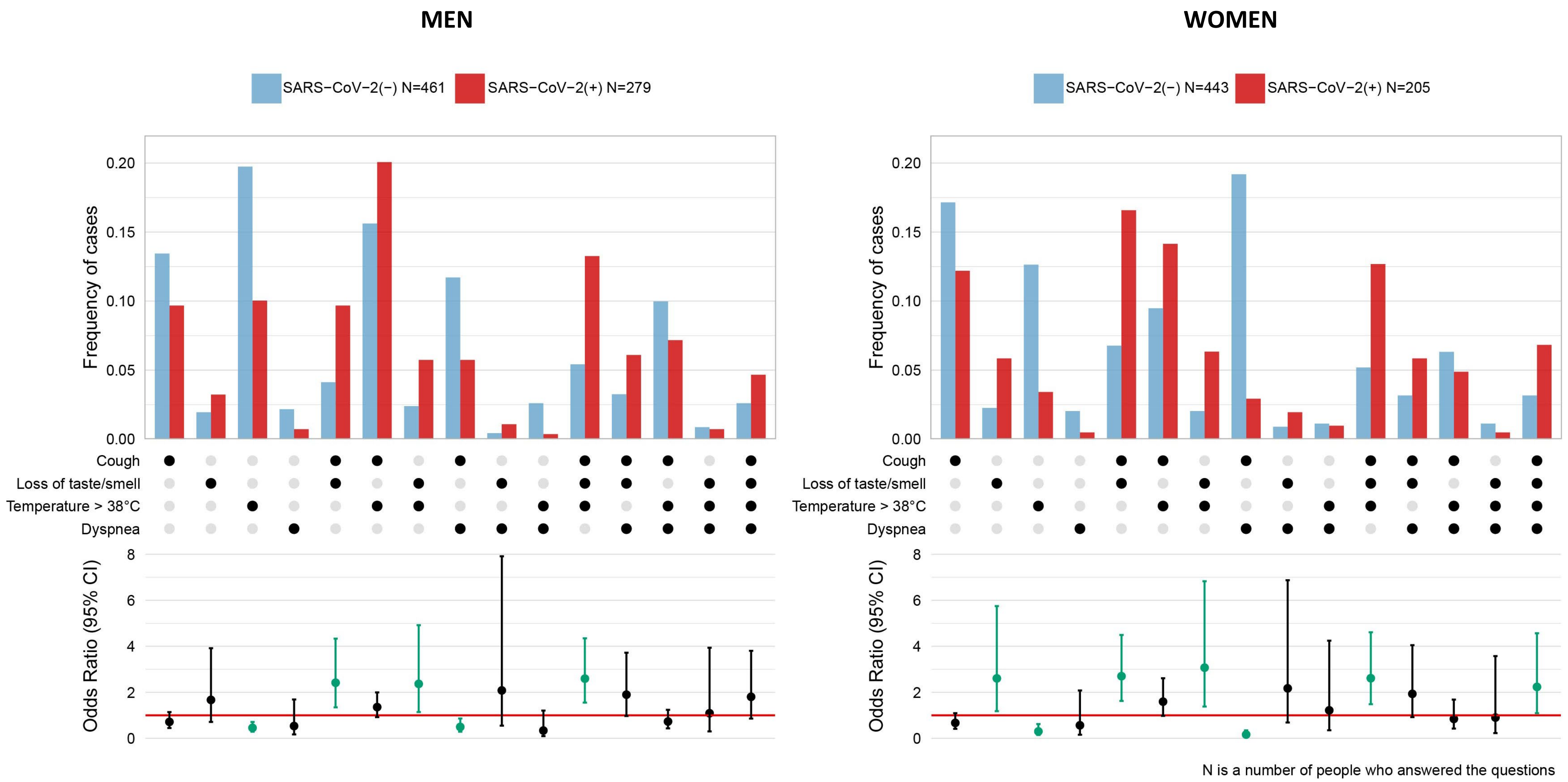
MEN
WOMEN
SARS−CoV−2(−) N=461
SARS−CoV−2(+) N=279
SARS−CoV−2(−) N=443
SARS−CoV−2(+) N=205
Frequency of cases
0.00
0.05
0.10
0.15
0.20
Cough
Loss of taste/smell
Temperature > 38°C
Dyspnea
Odds Ratio (95% CI)
0
2
4
6
8
N is a number of people who answered the questions

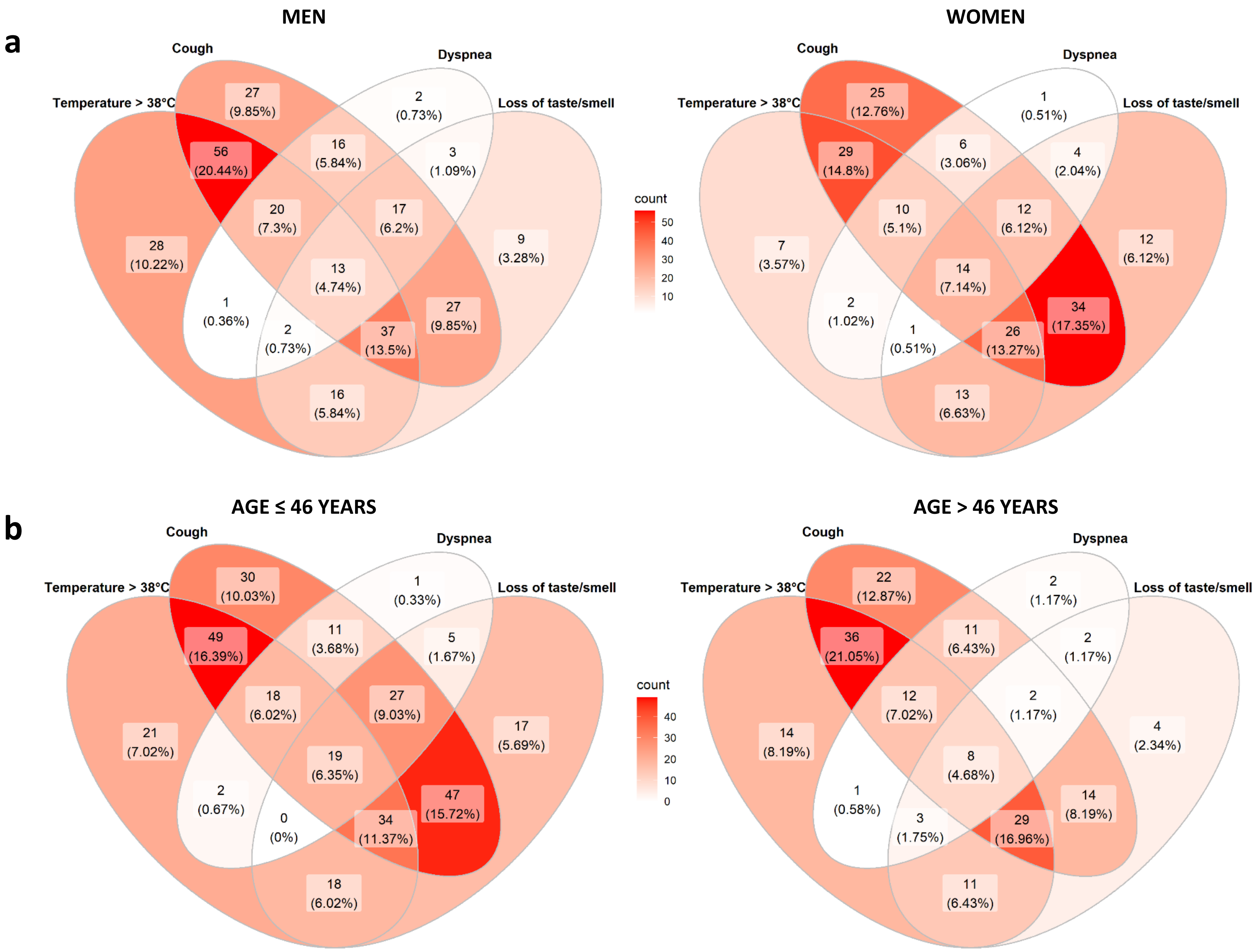
a
MEN
Cough
Dyspnea
Temperature > 38°C
Loss of taste/smell
27 (9.85%)
2 (0.73%)
56 (20.44%)
16 (5.84%)
3 (1.09%)
28 (10.22%)
20 (7.3%)
17 (6.2%)
9 (3.28%)
13 (4.74%)
1 (0.36%)
27 (9.85%)
2 (0.73%)
37 (13.5%)
16 (5.84%)
count
50
40
30
20
10
WOMEN
Cough
Dyspnea
Temperature > 38°C
Loss of taste/smell
25 (12.76%)
1 (0.51%)
29 (14.8%)
6 (3.06%)
4 (2.04%)
10 (5.1%)
12 (6.12%)
7 (3.57%)
12 (6.12%)
14 (7.14%)
2 (1.02%)
34 (17.35%)
1 (0.51%)
26 (13.27%)
13 (6.63%)
b
AGE ≤ 46 YEARS
Cough
Dyspnea
Temperature > 38°C
Loss of taste/smell
30 (10.03%)
1 (0.33%)
49 (16.39%)
11 (3.68%)
5 (1.67%)
18 (6.02%)
27 (9.03%)
21 (7.02%)
17 (5.69%)
19 (6.35%)
2 (0.67%)
47 (15.72%)
0 (0%)
34 (11.37%)
18 (6.02%)
count
40
30
20
10
0
AGE > 46 YEARS
Cough
Dyspnea
Temperature > 38°C
Loss of taste/smell
22 (12.87%)
2 (1.17%)
36 (21.05%)
11 (6.43%)
2 (1.17%)
12 (7.02%)
2 (1.17%)
14 (8.19%)
4 (2.34%)
8 (4.68%)
1 (0.58%)
14 (8.19%)
3 (1.75%)
29 (16.96%)
11 (6.43%)

Sample N = 3114

Continuous variables:

	N	Mean	SD	Median	Q1 [25%]	Q3 [75%]	Min	Max	no. of missing data
Age	3111	43,29	14,45	42	32	54	1	98	3
Saturation	3006	97,20	1,32	97	97	98	88	100	108
Temp. in hospital	2976	36,47	0,46	36,5	36,3	36,6	33,9	40,2	138
Max. temperature	1916	37,68	1,05	37,7	37	38,5	30,9	41,2	1198
Days of symptoms	1827	5,35	8,03	4	2	7	0	180	1287

Discrete variables:

	Yes	No	N	no. of missing data	Yes [% of N]	No [% of N]
Sex	1559	1554	3113	1	50,08	49,92
Number of infections	759	2355	3114	0	24,37	75,63
Health state	1941	1173	3114	0	62,33	37,67
Contact with infection	1227	1785	3012	102	40,74	59,26
Temperature > 38	931	2183	3114	0	29,90	70,10
Cough	1588	1526	3114	0	51,00	49,00
Dyspnea	724	2390	3114	0	23,25	76,75
Conjunctivitis	9	740	749	2365	1,20	98,80
Muscle aches	958	1288	2246	868	42,65	57,35
Loss of taste/smell	519	1725	2244	870	23,13	76,87
Sore throat	679	1566	2245	869	30,24	69,76
Tiredness	83	749	832	2282	9,98	90,02
Headache	971	1278	2249	865	43,17	56,83
Dizziness	348	1703	2051	1063	16,97	83,03
Skin reactions	60	2163	2223	891	2,70	97,30
Stomach symptoms	46	785	831	2283	5,54	94,46
Loss of appetite	8	762	770	2344	1,04	98,96
Lung disease	47	3067	3114	0	1,51	98,49
Hypertension	533	2581	3114	0	17,12	82,88
Heart diseases	163	2951	3114	0	5,23	94,77
Diabetes	133	2981	3114	0	4,27	95,73
Kidney diseases	34	3080	3114	0	1,09	98,91
Alergies	84	3030	3114	0	2,70	97,30
Asthma	133	2981	3114	0	4,27	95,73

**Blood type:**	n	% of N
0	470	39,13
A	407	33,89
AB	115	9,58
B	209	17,4
N	1201	100
no.of missing data	1913	

Symptoms considered in the classifier (in 10-fold cross-validation procedure)	Size of the training dataset N	Mean AUC (95%CI)	Mean DOR (95%CI)	Mean Sensitivity (95%CI)	Mean Specificity (95%CI)	Mean PPV (95%CI)	Mean NPV (95%CI)	Mean F1 (95%CI)	Mean Balanced Accuracy (95%CI)	Mean FPR (95%CI)	Mean FNR (95%CI)	Mean Youden (95%CI)
Temperature > 38°C	1 941	0.563 (0.532-0.595)	1.787 (1.282-2.292)	0.553 (0.501-0.605)	0.574 (0.533-0.615)	0.361 (0.330-0.391)	0.748 (0.724-0.772)	0.436 (0.401-0.471)	0.563 (0.532-0.595)	0.426 (0.385-0.467)	0.447 (0.395-0.499)	0.127 (0.064-0.190)
Cough	1 941	0.521 (0.499-0.544)	1.378 (0.974-1.781)	0.771 (0.729-0.813)	0.272 (0.248-0.296)	0.314 (0.301-0.327)	0.735 (0.695-0.774)	0.446 (0.427-0.465)	0.521 (0.499-0.544)	0.728 (0.704-0.752)	0.229 (0.187-0.271)	0.043 (-0.003-0.089)
Dyspnea	1 941	0.565 (0.551-0.579)	1.864 (1.625-2.103)	0.746 (0.724-0.767)	0.385 (0.358-0.411)	0.344 (0.334-0.354)	0.777 (0.762-0.792)	0.471 (0.459-0.482)	0.565 (0.551-0.579)	0.615 (0.589-0.642)	0.254 (0.233-0.276)	0.130 (0.103-0.158)
Conjunctivitis	349	0.519 (0.506-0.531)		1.000 (1.000-1.000)	0.037 (0.012-0.063)	0.477 (0.468-0.485)	1.000 (1.000-1.000)	0.645 (0.638-0.653)	0.519 (0.506-0.531)	0.963 (0.937-0.988)	0.000 (0.000-0.000)	0.037 (0.012-0.063)
Muscle aches	1 394	0.531 (0.505-0.557)	1.370 (1.018-1.723)	0.649 (0.615-0.683)	0.413 (0.380-0.446)	0.371 (0.350-0.391)	0.688 (0.658-0.718)	0.472 (0.448-0.496)	0.531 (0.505-0.557)	0.587 (0.554-0.620)	0.351 (0.317-0.385)	0.062 (0.009-0.115)
Loss of taste/smell	1 388	0.634 (0.603-0.665)	3.670 (2.494-4.847)	0.496 (0.450-0.542)	0.772 (0.739-0.805)	0.540 (0.496-0.585)	0.741 (0.719-0.763)	0.516 (0.475-0.557)	0.634 (0.603-0.665)	0.228 (0.195-0.261)	0.504 (0.458-0.550)	0.268 (0.205-0.331)
Sore throat	1 390	0.519 (0.484-0.555)	1.267 (0.917-1.616)	0.597 (0.539-0.655)	0.442 (0.397-0.487)	0.362 (0.334-0.389)	0.674 (0.636-0.712)	0.450 (0.414-0.485)	0.519 (0.484-0.555)	0.558 (0.513-0.603)	0.403 (0.345-0.461)	0.039 (-0.031-0.109)
Tiredness	419	0.542 (0.496-0.588)	3.037 (0.956-5.118)	0.876 (0.816-0.936)	0.209 (0.162-0.256)	0.464 (0.437-0.492)	0.701 (0.555-0.847)	0.607 (0.570-0.643)	0.542 (0.496-0.588)	0.791 (0.744-0.838)	0.124 (0.064-0.184)	0.085 (-0.007-0.177)
Headache	1 395	0.529 (0.489-0.569)	1.427 (0.968-1.887)	0.633 (0.572-0.695)	0.424 (0.388-0.460)	0.367 (0.338-0.396)	0.687 (0.639-0.735)	0.464 (0.426-0.503)	0.529 (0.489-0.569)	0.576 (0.540-0.612)	0.367 (0.305-0.428)	0.057 (-0.023-0.137)
Dizziness	1 317	0.519 (0.493-0.546)	1.332 (0.945-1.719)	0.777 (0.746-0.809)	0.261 (0.229-0.293)	0.364 (0.347-0.380)	0.682 (0.635-0.728)	0.495 (0.474-0.516)	0.519 (0.493-0.546)	0.739 (0.707-0.771)	0.223 (0.191-0.254)	0.038 (-0.014-0.091)
Skin reactions	1 374	0.498 (0.493-0.503)	0.940 (0.574-1.307)	0.127 (0.000-0.331)	0.869 (0.658-1.000)	0.326 (0.230-0.421)	0.635 (0.601-0.669)	0.109 (0.008-0.210)	0.498 (0.493-0.503)	0.131 (0.000-0.342)	0.873 (0.669-1.077)	-0.004 (-0.014-0.005)
GI symptoms	401	0.560 (0.534-0.585)	2.375 (0.000-4.943)	0.955 (0.909-1.000)	0.164 (0.115-0.213)	0.470 (0.456-0.484)	0.877 (0.756-0.999)	0.629 (0.610-0.648)	0.560 (0.534-0.585)	0.836 (0.787-0.885)	0.045 (0.000-0.091)	0.119 (0.067-0.171)
Loss of appetite	360	0.507 (0.492-0.523)	0.877 (0.000-4.821)	0.030 (0.000-0.060)	0.984 (0.959-1.000)	0.708 (0.162-1.000)	0.540 (0.533-0.546)	0.053 (0.000-0.107)	0.507 (0.492-0.523)	0.016 (0.000-0.041)	0.970 (0.940-1.000)	0.014 (-0.017-0.046)
Temperature > 38°C & Dyspnea	1 941	0.593 (0.570-0.615)	2.025 (1.448-2.601)	0.809 (0.755-0.862)	0.303 (0.243-0.363)	0.335 (0.324-0.346)	0.788 (0.757-0.819)	0.473 (0.458-0.487)	0.556 (0.538-0.574)	0.697 (0.637-0.757)	0.191 (0.138-0.245)	0.111 (0.075-0.148)
Temperature > 38°C & Cough	1 941	0.577 (0.549-0.605)	2.261 (1.569-2.953)	0.416 (0.373-0.460)	0.745 (0.704-0.785)	0.417 (0.374-0.461)	0.747 (0.730-0.764)	0.415 (0.377-0.453)	0.580 (0.552-0.609)	0.255 (0.215-0.296)	0.584 (0.540-0.627)	0.161 (0.104-0.218)
Temperature > 38°C & Muscle aches	1 394	0.540 (0.517-0.562)	1.156 (1.047-1.265)	0.465 (0.356-0.575)	0.566 (0.464-0.668)	0.363 (0.351-0.375)	0.668 (0.656-0.679)	0.399 (0.359-0.439)	0.516 (0.504-0.527)	0.434 (0.332-0.536)	0.535 (0.425-0.644)	0.031 (0.009-0.053)
Temperature > 38°C & Loss of taste/smell	1 388	0.659 (0.631-0.686)	3.670 (2.494-4.847)	0.496 (0.450-0.542)	0.772 (0.739-0.805)	0.540 (0.496-0.585)	0.741 (0.719-0.763)	0.516 (0.475-0.557)	0.634 (0.603-0.665)	0.228 (0.195-0.261)	0.504 (0.458-0.550)	0.268 (0.205-0.331)
Temperature > 38°C & GI symptoms	401	0.602 (0.555-0.649)	2.586 (0.961-4.211)	0.553 (0.423-0.683)	0.597 (0.523-0.671)	0.509 (0.448-0.570)	0.643 (0.575-0.712)	0.523 (0.433-0.614)	0.575 (0.512-0.639)	0.403 (0.329-0.477)	0.447 (0.317-0.577)	0.151 (0.024-0.277)
Dyspnea & Cough	1 941	0.585 (0.569-0.600)	1.838 (1.554-2.123)	0.550 (0.514-0.585)	0.595 (0.567-0.623)	0.370 (0.352-0.389)	0.753 (0.738-0.769)	0.442 (0.420-0.464)	0.572 (0.553-0.592)	0.405 (0.377-0.433)	0.450 (0.415-0.486)	0.144 (0.105-0.184)
Dyspnea & Muscle aches	1 394	0.562 (0.536-0.589)	1.788 (1.125-2.450)	0.744 (0.695-0.793)	0.353 (0.314-0.392)	0.380 (0.365-0.394)	0.724 (0.681-0.766)	0.502 (0.481-0.523)	0.548 (0.525-0.572)	0.647 (0.608-0.686)	0.256 (0.207-0.305)	0.097 (0.050-0.144)
Dyspnea & Loss of taste/smell	1 388	0.661 (0.626-0.696)	3.670 (2.494-4.847)	0.496 (0.450-0.542)	0.772 (0.739-0.805)	0.540 (0.496-0.585)	0.741 (0.719-0.763)	0.516 (0.475-0.557)	0.634 (0.603-0.665)	0.228 (0.195-0.261)	0.504 (0.458-0.550)	0.268 (0.205-0.331)
Dyspnea & GI symptoms	401	0.573 (0.522-0.623)	1.769 (1.043-2.496)	0.717 (0.593-0.842)	0.368 (0.260-0.476)	0.467 (0.436-0.499)	0.672 (0.572-0.771)	0.560 (0.507-0.613)	0.543 (0.505-0.581)	0.632 (0.524-0.740)	0.283 (0.158-0.407)	0.086 (0.010-0.161)
Cough & Muscle aches	1 394	0.560 (0.523-0.597)	1.626 (1.184-2.068)	0.496 (0.451-0.541)	0.608 (0.558-0.657)	0.404 (0.371-0.437)	0.693 (0.668-0.718)	0.444 (0.410-0.478)	0.552 (0.521-0.583)	0.392 (0.343-0.442)	0.504 (0.459-0.549)	0.104 (0.042-0.165)
Cough & Loss of taste/smell	1 388	0.656 (0.617-0.694)	3.670 (2.494-4.847)	0.496 (0.450-0.542)	0.772 (0.739-0.805)	0.540 (0.496-0.585)	0.741 (0.719-0.763)	0.516 (0.475-0.557)	0.634 (0.603-0.665)	0.228 (0.195-0.261)	0.504 (0.458-0.550)	0.268 (0.205-0.331)
Cough & GI symptoms	401	0.602 (0.551-0.653)	2.835 (1.602-4.067)	0.743 (0.678-0.809)	0.431 (0.350-0.511)	0.505 (0.466-0.545)	0.683 (0.603-0.763)	0.600 (0.558-0.642)	0.587 (0.536-0.638)	0.569 (0.489-0.650)	0.257 (0.191-0.322)	0.174 (0.071-0.277)
Muscle aches & Loss of taste/smell	1 379	0.645 (0.610-0.681)	3.774 (2.666-4.883)	0.493 (0.432-0.554)	0.777 (0.734-0.820)	0.547 (0.499-0.594)	0.742 (0.717-0.766)	0.515 (0.468-0.562)	0.635 (0.601-0.669)	0.223 (0.180-0.266)	0.507 (0.446-0.568)	0.270 (0.203-0.337)
Muscle aches & GI symptoms	399	0.606 (0.532-0.680)	2.988 (1.282-4.694)	0.681 (0.602-0.760)	0.501 (0.417-0.585)	0.518 (0.460-0.576)	0.665 (0.588-0.742)	0.587 (0.524-0.651)	0.591 (0.523-0.658)	0.499 (0.415-0.583)	0.319 (0.240-0.398)	0.182 (0.047-0.316)
Loss of taste/smell & GI symptoms	398	0.645 (0.588-0.702)	5.004 (1.959-8.050)	0.402 (0.319-0.485)	0.829 (0.771-0.888)	0.653 (0.552-0.754)	0.640 (0.597-0.683)	0.493 (0.407-0.578)	0.616 (0.558-0.674)	0.171 (0.112-0.229)	0.598 (0.515-0.681)	0.231 (0.115-0.347)
Temperature > 38°C & Dyspnea & Cough	1 941	0.602 (0.580-0.623)	1.879 (1.470-2.288)	0.479 (0.288-0.670)	0.620 (0.425-0.816)	0.383 (0.334-0.433)	0.744 (0.722-0.767)	0.383 (0.327-0.438)	0.550 (0.535-0.565)	0.380 (0.184-0.575)	0.521 (0.330-0.712)	0.099 (0.069-0.129)
Temperature > 38°C & Dyspnea & Muscle aches	1 394	0.563 (0.536-0.590)	1.594 (1.325-1.862)	0.744 (0.676-0.812)	0.345 (0.263-0.427)	0.378 (0.366-0.391)	0.716 (0.684-0.748)	0.499 (0.483-0.515)	0.544 (0.526-0.563)	0.655 (0.573-0.737)	0.256 (0.188-0.324)	0.089 (0.053-0.125)
Temperature > 38°C & Dyspnea & Loss of taste/smell	1 388	0.671 (0.639-0.702)	3.670 (2.494-4.847)	0.496 (0.450-0.542)	0.772 (0.739-0.805)	0.540 (0.496-0.585)	0.741 (0.719-0.763)	0.516 (0.475-0.557)	0.634 (0.603-0.665)	0.228 (0.195-0.261)	0.504 (0.458-0.550)	0.268 (0.205-0.331)
Temperature > 38°C & Dyspnea & GI symptoms	401	0.588 (0.522-0.654)	2.333 (0.476-4.189)	0.632 (0.443-0.821)	0.437 (0.292-0.582)	0.460 (0.420-0.500)	0.640 (0.553-0.727)	0.515 (0.426-0.604)	0.535 (0.488-0.582)	0.563 (0.418-0.708)	0.368 (0.179-0.557)	0.070 (-0.025-0.164)
Temperature > 38°C & Cough & Muscle aches	1 394	0.582 (0.550-0.613)	1.743 (1.291-2.195)	0.457 (0.374-0.540)	0.658 (0.563-0.753)	0.421 (0.387-0.455)	0.693 (0.668-0.718)	0.431 (0.387-0.475)	0.558 (0.527-0.588)	0.342 (0.247-0.437)	0.543 (0.460-0.626)	0.115 (0.054-0.176)
Temperature > 38°C & Cough & Loss of taste/smell	1 388	0.672 (0.641-0.703)	3.863 (2.769-4.957)	0.733 (0.697-0.770)	0.564 (0.532-0.597)	0.475 (0.451-0.499)	0.798 (0.772-0.824)	0.576 (0.550-0.602)	0.649 (0.622-0.676)	0.436 (0.403-0.468)	0.267 (0.230-0.303)	0.298 (0.244-0.351)
Temperature > 38°C & Cough & GI symptoms	401	0.642 (0.591-0.693)	2.288 (1.189-3.386)	0.461 (0.298-0.624)	0.647 (0.477-0.818)	0.527 (0.447-0.607)	0.615 (0.556-0.675)	0.462 (0.379-0.545)	0.554 (0.507-0.602)	0.353 (0.182-0.523)	0.539 (0.376-0.702)	0.109 (0.015-0.203)
Temperature > 38°C & Muscle aches & Loss of taste/smell	1 379	0.664 (0.626-0.703)	3.774 (2.666-4.883)	0.493 (0.432-0.554)	0.777 (0.734-0.820)	0.547 (0.499-0.594)	0.742 (0.717-0.766)	0.515 (0.468-0.562)	0.635 (0.601-0.669)	0.223 (0.180-0.266)	0.507 (0.446-0.568)	0.270 (0.203-0.337)
Temperature > 38°C & Muscle aches & GI symptoms	399	0.611 (0.532-0.691)	1.959 (1.021-2.897)	0.652 (0.525-0.779)	0.442 (0.364-0.519)	0.471 (0.425-0.518)	0.635 (0.562-0.708)	0.542 (0.468-0.616)	0.547 (0.494-0.599)	0.558 (0.481-0.636)	0.348 (0.221-0.475)	0.093 (-0.012-0.199)
Temperature > 38°C & Loss of taste/smell & GI symptoms	398	0.681 (0.612-0.749)	4.303 (1.806-6.799)	0.731 (0.598-0.864)	0.521 (0.401-0.642)	0.553 (0.502-0.603)	0.739 (0.638-0.840)	0.618 (0.549-0.686)	0.626 (0.569-0.683)	0.479 (0.358-0.599)	0.269 (0.136-0.402)	0.252 (0.138-0.366)
Dyspnea & Cough & Muscle aches	1 394	0.588 (0.563-0.612)	1.930 (1.567-2.292)	0.540 (0.483-0.596)	0.613 (0.591-0.635)	0.425 (0.405-0.444)	0.716 (0.693-0.739)	0.474 (0.442-0.507)	0.576 (0.553-0.600)	0.387 (0.365-0.409)	0.460 (0.404-0.517)	0.153 (0.106-0.200)
Dyspnea & Cough & Loss of taste/smell	1 388	0.679 (0.639-0.718)	3.416 (1.849-4.983)	0.712 (0.609-0.816)	0.523 (0.418-0.627)	0.454 (0.417-0.491)	0.780 (0.741-0.818)	0.544 (0.511-0.576)	0.617 (0.589-0.646)	0.477 (0.373-0.582)	0.288 (0.184-0.391)	0.235 (0.178-0.291)
Dyspnea & Cough & GI symptoms	401	0.625 (0.567-0.682)	2.090 (1.268-2.913)	0.546 (0.420-0.672)	0.581 (0.477-0.685)	0.504 (0.454-0.554)	0.635 (0.577-0.693)	0.514 (0.445-0.583)	0.564 (0.519-0.608)	0.419 (0.315-0.523)	0.454 (0.328-0.580)	0.128 (0.038-0.217)
Dyspnea & Muscle aches & Loss of taste/smell	1 379	0.665 (0.631-0.700)	3.774 (2.666-4.883)	0.493 (0.432-0.554)	0.777 (0.734-0.820)	0.547 (0.499-0.594)	0.742 (0.717-0.766)	0.515 (0.468-0.562)	0.635 (0.601-0.669)	0.223 (0.180-0.266)	0.507 (0.446-0.568)	0.270 (0.203-0.337)
Dyspnea & Muscle aches & GI symptoms	399	0.616 (0.541-0.690)	2.179 (1.174-3.183)	0.548 (0.473-0.623)	0.585 (0.476-0.695)	0.520 (0.459-0.581)	0.619 (0.563-0.676)	0.527 (0.476-0.578)	0.567 (0.512-0.622)	0.415 (0.305-0.524)	0.452 (0.377-0.527)	0.134 (0.023-0.244)
Dyspnea & Loss of taste/smell & GI symptoms	398	0.639 (0.580-0.698)	5.004 (1.959-8.050)	0.402 (0.319-0.485)	0.829 (0.771-0.888)	0.653 (0.552-0.754)	0.640 (0.597-0.683)	0.493 (0.407-0.578)	0.616 (0.558-0.674)	0.171 (0.112-0.229)	0.598 (0.515-0.681)	0.231 (0.115-0.347)
Cough & Muscle aches & Loss of taste/smell	1 379	0.664 (0.632-0.696)	3.774 (2.666-4.883)	0.493 (0.432-0.554)	0.777 (0.734-0.820)	0.547 (0.499-0.594)	0.742 (0.717-0.766)	0.515 (0.468-0.562)	0.635 (0.601-0.669)	0.223 (0.180-0.266)	0.507 (0.446-0.568)	0.270 (0.203-0.337)
Cough & Muscle aches & GI symptoms	399	0.602 (0.530-0.674)	1.868 (0.859-2.877)	0.636 (0.533-0.739)	0.444 (0.308-0.581)	0.479 (0.423-0.536)	0.587 (0.469-0.704)	0.540 (0.481-0.599)	0.540 (0.477-0.604)	0.556 (0.419-0.692)	0.364 (0.261-0.467)	0.081 (-0.046-0.207)
Cough & Loss of taste/smell & GI symptoms	398	0.689 (0.630-0.748)	4.582 (2.000-7.163)	0.842 (0.714-0.970)	0.412 (0.288-0.536)	0.533 (0.496-0.570)	0.804 (0.705-0.902)	0.643 (0.584-0.703)	0.627 (0.577-0.677)	0.588 (0.464-0.712)	0.158 (0.030-0.286)	0.254 (0.154-0.355)
Muscle aches & Loss of taste/smell & GI symptoms	398	0.683 (0.620-0.747)	5.218 (2.389-8.046)	0.819 (0.755-0.882)	0.448 (0.381-0.515)	0.540 (0.506-0.573)	0.763 (0.694-0.832)	0.649 (0.611-0.687)	0.633 (0.590-0.677)	0.552 (0.485-0.619)	0.181 (0.118-0.245)	0.267 (0.180-0.353)
Temperature > 38°C & Dyspnea & Cough & Muscle aches	1 394	0.599 (0.577-0.621)	1.862 (1.552-2.173)	0.585 (0.517-0.654)	0.559 (0.489-0.630)	0.416 (0.395-0.436)	0.718 (0.700-0.737)	0.483 (0.456-0.510)	0.572 (0.551-0.593)	0.441 (0.370-0.511)	0.415 (0.346-0.483)	0.144 (0.103-0.186)
Temperature > 38°C & Dyspnea & Cough & Loss of taste/smell	1 388	0.686 (0.650-0.723)	4.308 (2.645-5.970)	0.671 (0.623-0.720)	0.646 (0.600-0.692)	0.507 (0.465-0.548)	0.786 (0.755-0.817)	0.576 (0.536-0.617)	0.659 (0.621-0.696)	0.354 (0.308-0.400)	0.329 (0.280-0.377)	0.318 (0.242-0.393)
Temperature > 38°C & Dyspnea & Cough & GI symptoms	401	0.642 (0.585-0.699)	2.325 (0.636-4.014)	0.478 (0.319-0.637)	0.633 (0.517-0.749)	0.502 (0.446-0.558)	0.624 (0.556-0.692)	0.470 (0.375-0.565)	0.556 (0.502-0.610)	0.367 (0.251-0.483)	0.522 (0.363-0.681)	0.111 (0.004-0.219)
Temperature > 38°C & Dyspnea & Muscle aches & Loss of taste/smell	1 379	0.670 (0.634-0.706)	3.774 (2.666-4.883)	0.493 (0.432-0.554)	0.777 (0.734-0.820)	0.547 (0.499-0.594)	0.742 (0.717-0.766)	0.515 (0.468-0.562)	0.635 (0.601-0.669)	0.223 (0.180-0.266)	0.507 (0.446-0.568)	0.270 (0.203-0.337)
Temperature > 38°C & Dyspnea & Muscle aches & GI symptoms	399	0.618 (0.538-0.699)	2.435 (1.334-3.536)	0.618 (0.523-0.713)	0.540 (0.451-0.628)	0.515 (0.467-0.562)	0.648 (0.579-0.716)	0.557 (0.499-0.614)	0.579 (0.525-0.633)	0.460 (0.372-0.549)	0.382 (0.287-0.477)	0.157 (0.050-0.265)
Temperature > 38°C & Dyspnea & Loss of taste/smell & GI symptoms	398	0.671 (0.602-0.739)	4.787 (2.338-7.236)	0.725 (0.598-0.852)	0.521 (0.401-0.642)	0.551 (0.500-0.602)	0.728 (0.641-0.815)	0.615 (0.548-0.681)	0.623 (0.567-0.679)	0.479 (0.358-0.599)	0.275 (0.148-0.402)	0.247 (0.135-0.358)
Temperature > 38°C & Cough & Muscle aches & Loss of taste/smell	1 379	0.676 (0.641-0.711)	3.346 (2.619-4.073)	0.678 (0.626-0.730)	0.596 (0.527-0.665)	0.478 (0.444-0.513)	0.776 (0.753-0.799)	0.558 (0.531-0.585)	0.637 (0.609-0.665)	0.404 (0.335-0.473)	0.322 (0.270-0.374)	0.274 (0.218-0.329)
Temperature > 38°C & Cough & Muscle aches & GI symptoms	399	0.613 (0.533-0.693)	2.234 (0.351-4.117)	0.531 (0.404-0.658)	0.567 (0.457-0.677)	0.490 (0.427-0.552)	0.610 (0.541-0.679)	0.500 (0.415-0.585)	0.549 (0.485-0.613)	0.433 (0.323-0.543)	0.469 (0.342-0.596)	0.098 (-0.030-0.226)
Temperature > 38°C & Cough & Loss of taste/smell & GI symptoms	398	0.704 (0.632-0.776)	8.991 (3.792-14.191)	0.704 (0.578-0.831)	0.659 (0.581-0.738)	0.620 (0.548-0.692)	0.757 (0.667-0.846)	0.653 (0.569-0.737)	0.682 (0.612-0.752)	0.341 (0.262-0.419)	0.296 (0.169-0.422)	0.364 (0.223-0.504)
Temperature > 38°C & Muscle aches & Loss of taste/smell & GI symptoms	398	0.695 (0.624-0.766)	5.306 (2.608-8.004)	0.676 (0.576-0.777)	0.637 (0.556-0.717)	0.596 (0.526-0.666)	0.720 (0.654-0.786)	0.630 (0.553-0.706)	0.657 (0.591-0.722)	0.363 (0.283-0.444)	0.324 (0.223-0.424)	0.313 (0.183-0.443)
Dyspnea & Cough & Muscle aches & Loss of taste/smell	1 379	0.678 (0.648-0.708)	3.349 (2.425-4.273)	0.634 (0.561-0.708)	0.635 (0.580-0.690)	0.484 (0.455-0.513)	0.767 (0.736-0.799)	0.546 (0.509-0.582)	0.635 (0.605-0.664)	0.365 (0.310-0.420)	0.366 (0.292-0.439)	0.269 (0.209-0.329)
Dyspnea & Cough & Muscle aches & GI symptoms	399	0.619 (0.549-0.690)	1.808 (0.668-2.948)	0.599 (0.473-0.725)	0.455 (0.266-0.645)	0.487 (0.420-0.554)	0.558 (0.442-0.674)	0.516 (0.463-0.569)	0.527 (0.467-0.588)	0.545 (0.355-0.734)	0.401 (0.275-0.527)	0.055 (-0.067-0.176)
Dyspnea & Cough & Loss of taste/smell & GI symptoms	398	0.705 (0.640-0.769)	5.566 (2.806-8.327)	0.725 (0.652-0.799)	0.601 (0.532-0.670)	0.590 (0.532-0.649)	0.738 (0.671-0.805)	0.649 (0.590-0.709)	0.663 (0.603-0.723)	0.399 (0.330-0.468)	0.275 (0.201-0.348)	0.326 (0.207-0.446)
Dyspnea & Muscle aches & Loss of taste/smell & GI symptoms	398	0.683 (0.613-0.753)	3.443 (1.774-5.112)	0.670 (0.582-0.757)	0.561 (0.481-0.640)	0.548 (0.495-0.601)	0.691 (0.626-0.755)	0.599 (0.543-0.655)	0.615 (0.562-0.668)	0.439 (0.360-0.519)	0.330 (0.243-0.418)	0.230 (0.124-0.337)
Cough & Muscle aches & Loss of taste/smell & GI symptoms	398	0.711 (0.647-0.776)	5.738 (3.045-8.430)	0.744 (0.651-0.838)	0.574 (0.496-0.652)	0.580 (0.527-0.634)	0.749 (0.676-0.821)	0.649 (0.588-0.709)	0.659 (0.602-0.716)	0.426 (0.348-0.504)	0.256 (0.162-0.349)	0.318 (0.203-0.432)

LEGEND:

	panel of symptoms with estimated DOR>2 and AUC>65%
	panel of symptoms with estimated DOR>3 and AUC>70%

	All			Women			Men			Young			Old		
characteristics	Odds Ratio	95% Confidence Interval - Lower Bound	95% Confidence Interval - Upper Bound	Odds Ratio	95% Confidence Interval - Lower Bound	95% Confidence Interval - Upper Bound	Odds Ratio	95% Confidence Interval - Lower Bound	95% Confidence Interval - Upper Bound	Odds Ratio	95% Confidence Interval - Lower Bound	95% Confidence Interval - Upper Bound	Odds Ratio	95% Confidence Interval - Lower Bound	95% Confidence Interval - Upper Bound
Sex *	0,870	0,710	1,050	-	-	-	-	-	-	0,729	0,567	0,937	1,102	0,806	1,505
Contact with infected person	1,790	1,450	2,210	1,650	1,220	2,240	1,970	1,470	2,660	1,890	1,440	2,460	1,680	1,190	2,370
Temperature > 38oC	1,670	1,370	2,030	1,780	1,340	2,370	1,530	1,170	2,010	1,530	1,190	1,960	1,930	1,400	2,650
Cough	1,260	1,000	1,580	1,120	0,800	1,570	1,410	1,040	1,920	1,140	0,850	1,540	1,480	1,040	2,110
Dyspnea	0,550	0,440	0,680	0,520	0,380	0,710	0,580	0,430	0,770	0,560	0,430	0,730	0,530	0,370	0,770
Conjunctivitis	0,240	0,050	1,140	0,550	0,110	2,910	0,310	0,060	1,660	0,280	0,060	1,410	0,630	0,110	3,670
Muscle aches	1,300	1,030	1,630	1,200	0,850	1,690	1,420	1,040	1,920	1,730	1,280	2,350	0,860	0,600	1,230
Loss of taste/smell	3,330	2,630	4,220	3,990	2,810	5,670	3,000	2,170	4,160	3,900	2,890	5,260	2,560	1,730	3,790
Sore throat	0,850	0,680	1,070	0,660	0,470	0,920	1,130	0,830	1,540	0,700	0,530	0,930	1,210	0,830	1,760
Tiredness	0,540	0,320	0,930	0,730	0,350	1,550	0,490	0,230	1,030	0,570	0,290	1,100	0,500	0,190	1,270
Headache	1,270	1,010	1,590	1,150	0,810	1,640	1,440	1,060	1,940	1,410	1,040	1,900	1,060	0,740	1,530
Dizziness	0,810	0,620	1,060	0,980	0,660	1,440	0,700	0,490	1,010	0,700	0,500	0,980	1,060	0,680	1,640
Skin reactions	1,080	0,630	1,880	1,230	0,590	2,570	1,060	0,490	2,300	1,110	0,550	2,260	1,150	0,520	2,580
GI symptoms	0,290	0,140	0,600	0,470	0,190	1,160	0,250	0,090	0,710	0,290	0,120	0,700	0,400	0,140	1,170
Loss of appetite	1,650	0,510	5,330	1,730	0,470	6,270	1,780	0,310	10,210	1,390	0,360	5,380	1,730	0,360	8,170
Lung disease	0,440	0,180	1,050	0,640	0,210	1,980	0,460	0,150	1,390	1,190	0,290	4,860	0,380	0,140	1,000
Hypertension	0,910	0,700	1,170	0,940	0,640	1,360	0,880	0,620	1,250	0,790	0,490	1,260	0,910	0,650	1,260
Heart diseases	0,640	0,410	1,000	0,490	0,250	0,950	0,900	0,500	1,620	0,860	0,400	1,860	0,560	0,330	0,960
Diabetes	0,750	0,460	1,200	0,560	0,260	1,240	0,930	0,530	1,650	0,790	0,310	2,020	0,730	0,430	1,240
Kidney diseases	0,960	0,420	2,220	1,230	0,410	3,660	0,960	0,330	2,820	1,190	0,290	4,860	0,990	0,400	2,480
Allergies	0,460	0,230	0,910	0,450	0,180	1,090	0,660	0,260	1,690	0,500	0,220	1,140	0,580	0,210	1,590
Asthma	0,680	0,420	1,110	0,810	0,430	1,510	0,630	0,310	1,300	0,460	0,230	0,930	1,210	0,620	2,370

**LEGEND:**	
(green)	significant odds ratio higher than 1 (potential risk factor)
(red)	significant odds ratio smaller than 1 (potential protective factor)
